# Tumor stem-like cell-derived exosomal RNAs prime neutrophils for facilitating tumorigenesis of colon cancer

**DOI:** 10.1186/s13045-019-0699-4

**Published:** 2019-01-25

**Authors:** Wei-Lun Hwang, Hsin-Yi Lan, Wei-Chung Cheng, Shih-Ching Huang, Muh-Hwa Yang

**Affiliations:** 1 Department of Biotechnology and Laboratory Science in Medicine, National Yang-Ming University, No. 155, Sec. 2, Li-Nong St., Taipei, 11221 Taiwan; 2 The Ph.D. Program for Translational Medicine, College of Medical Science and Technology, Taipei Medical University, No.250, Wuxing St., Taipei, 11031 Taiwan; 3 Cancer Progression Research Center, National Yang-Ming University, No. 155, Sec. 2, Li-Nong Street, Taipei, 11221 Taiwan; 4 Institute of Clinical Medicine, National Yang-Ming University, No. 155, Sec. 2, Li-Nong Street, Taipei, 11221 Taiwan; 5 Graduate Institute of Biomedical Science, Research Center for Tumor Medical Science, and Drug Development Center, China Medical University, No.6, Hsueh-Shih Road, Taichung, 40403 Taiwan; 6 Division of Medical Oncology, Department of Oncology, Taipei Veterans General Hospital, No.201, Sec. 2, Shipai Rd., Taipei, 11217 Taiwan

**Keywords:** Tumor-host interaction, Cancer stem cells, Neutrophils, Exosomes, Interleukin-1β

## Abstract

**Background:**

Cell-cell interactions maintain tissue homeostasis and contribute to dynamic alteration of the tumor microenvironment (TME). Communication between cancer and host cells not only promotes advanced disease aggression but also determines therapeutic response in cancer patients. Despite accumulating evidence supporting the role of tumor-infiltrating immunocytes in modulating tumor immunity, the interplay between heterogeneous tumor subpopulations and immunocytes is elusive.

**Methods:**

We expanded colorectal cancer stem cells (CRCSCs) as cancer spheroids from the murine colorectal cancer (CRC) cell line CT26 to interrogate tumor-host interactions using a syngeneic tumor model. RNA-sequencing analysis of host cells and tumor exosomes was performed to identify molecular determinants that mediate the crosstalk between CRCSCs and immunocytes. The Cancer Genome Atlas (TCGA) database was used to validate the clinical significance in CRC patients.

**Results:**

The expanded CT26 cancer spheroids showed increased stemness gene expression, enhanced spheroid and clonogenicity potential, and an elevated tumor-initiating ability, characteristic of CRCSCs. By examining immune cell composition in syngeneic tumor-bearing mice, a systemic increase in CD11b^+^/Ly6G^High^/Ly6C^Low^ neutrophils was observed in mice bearing CRCSC-derived tumors. An increased secretion of CRCSC exosomes was observed in vitro, and through in vivo tracking, CRCSC exosomes were found to be transported to the bone marrow. Moreover, CRCSC exosomes prolonged the survival of bone marrow-derived neutrophils and engendered a protumoral phenotype in neutrophils. Mechanistically, tumor exosomal tri-phosphate RNAs induced the expression of interleukin-1β (IL-1β) through a pattern recognition-NF-κB signaling axis to sustain neutrophil survival. CRCSC-secreted CXCL1 and CXCL2 then attracted CRCSC-primed neutrophils to promote tumorigenesis of CRC cells via IL-1β. Moreover, neutrophil depletion using a Ly6G-specific antibody (clone 1A8) attenuated the tumorigenicity of CRCSCs. In human specimens, CRC patients exhibiting an active CRCSC signal (Snail^+^IL8^+^) showed elevated tumor infiltration of MPO^+^ neutrophils, and high (in the top 10%) MPO expression predicted poor survival of CRC patients.

**Conclusions:**

This study elucidates a multistep CRCSC-neutrophil interaction during advanced cancer progression. Strategies targeting aberrant neutrophil activation may be developed for combating CSC-related malignancy.

**Electronic supplementary material:**

The online version of this article (10.1186/s13045-019-0699-4) contains supplementary material, which is available to authorized users.

## Background

Tumors are highly heterogeneous tissues composed of tumor cells and diverse host cells, including tumor-infiltrating immune cells, endothelial cells, and fibroblasts [1]. The intratumor heterogeneity arising from clonal alteration and cell plasticity drives dynamic cell-cell communication and ultimately leads to a heterogeneous therapeutic response during cancer progression [2, 3]. The intestinal epithelium is a monolayer of cells organized into crypts and villi and is known as the most rapidly renewing tissue in adult mammals [4]. Though homeostatic stem cell cycling participates in tissue maintenance, loss of APC in Lgr5+ intestinal stem cells (ISCs) and aberrant expansion of intestinal progenitors induced by a high-fat diet potentiate early cancer development [5, 6]. Cancer stem cells (CSCs), which may evolve from normal counterparts, represent a small population of cancer cells with stem cell functions responsible for tumor initiation, therapeutic resistance, and distant metastasis [7, 8]. The expression of an ISC signature and CSC signature is a prognostic factor indicating a poor clinical outcome in cancer patients [9, 10]. Therefore, targeting cancerous stem cell-host interactions is important for combating tumor development and cancer progression.

Host myeloid cells are a physiological barrier against pathogen infection and are critical for tumorigenesis and cancer metastasis [11, 12]. Compared with other immune cells, neutrophils are particularly crucial for tumor initiation but have attracted little attention because of their limited lifespan [13]. Neutrophils not only support early tumorigenesis by inducing angiogenesis through MMP-9 [14] but also accelerate tumor proliferation by delivering neutrophil elastase [15]. However, mechanisms explaining granulopoiesis and neutrophil survival are limited.

Exosomes are microvesicles generated from intraluminal vesicles (ILVs) of multivescular bodies (MVBs) and are released from almost all cells for intercellular communication [16]. Exosomes containing adhesion molecules, cholesterols, and nucleic acids are distinct from platelet-derived microparticles [17]. The orchestration of premetastatic niches by organ-specific tumor exosome targeting further suggests a systemic effect of tumor exosomes on the host [18]. We previously showed that colorectal cancer stem cells (CRCSCs) had undergone epithelial-mesenchymal transition (EMT) and demonstrated that the Snail-IL8 axis elicited angiogenesis and cancer stemness in the tumor microenvironment [19]. In this study, we uncover the mechanism governing CRCSC-regulated neutrophil expansion and demonstrate the impacts of exosomes on the CRCSC-neutrophil interaction during cancer progression.

## Methods

### Cell culture and expansion of CRCSCs

The murine CT26 and 293 T cells were cultured in DMEM (Gibco) supplemented with 10% fetal bovine serum (FBS, Gibco). The CRCSCs were enriched as cancer spheroids by culturing cells in serum-free, stem cell medium (SCM) prepared with DMEM/F12 (Gibco) plus 10 ng/ml EGF (PeproTech), 10 ng/ml bFGF (PeproTech), and N2 supplements (Gibco) for 2 weeks. The resultant spheroids were defined as sphere-derived cancer stem cells (SDCSCs), and cells were dissociated with TryPLE Express (Gibco) for counting and further experiments. The sorted murine immune cells were grown in RPMI-1640 (Gibco) supplemented with 10% FBS. All cells were cultured in a humidified incubator at 37 °C under 5% CO_2_.

### Sphere-forming and clonogenicity assay

A total of 1000 cells were suspended per well in a 96-well plate under defined stem cell medium cultivation for 7 days, and the number of spheroids larger than 50 μm was counted by visual inspection. In IL-1β-depleted spheroid formation experiments, CT26 cells were cultured under neutrophil conditioned medium in the presence of 10 μg/ml IL-1β neutralizing antibody (AF-401-NA; R&D Systems Inc.) or 10 μg/ml normal goat IgG control (AB-108-C, R&D System Inc.) for 3 days prior to the sphere-forming assay. For colony formation assays, 1000 cells were seeded per well in a 6-well plate in complete DMEM for 7 days. Cells were fixed with 4% of paraformaldehyde (Sigma-Aldrich) and stained with 0.05% of crystal violet (Sigma-Aldrich) for counting colonies.

### Purification of tumor exosomes

Day 14–21 cancer spheroid medium was collected and centrifuged at 2000*g*, 4 °C for 15 min followed by 10,000*g*, 4 °C for 30 min to remove cell debris. The supernatants were subjected for one additional ultracentrifugation at 100,000*g*, 4 °C for 1.5 h to pellet exosomes in an Optima_L90-K ultracentrifuge (Beckman Coulter). The exosome pellets were resuspended and washed with DPBS (Gibco) twice by ultracentrifugation at 100,000*g*, 4 °C for 1.5 h using an Optima TLX ultracentrifuge (Beckman Coulter). To harvest parental CRC cell-secreted exosomes, complete medium containing 10% exosome-depleted FBS was prepared prior to tumor exosome collection. The exosome pellets were then resuspended in DPBS for in vitro and in vivo characterization, RIPA lysis buffer (50 mM Tris-HCL pH = 8, 150 mM NaCl, 0.5% sodium deoxycholate and 0.1% SDS) for protein quantification, or miRNeasy mini kit (Qiagen) reagent for RNA extraction.

### Transmission electron microscopy (TEM) and nanoparticle tracking analysis (NTA)

For TEM, exosomes were fixed in 2% paraformaldehyde (Sigma-Aldrich) and spread onto carbon/Formvar-coated grid (Ted Pella, Inc.) for 30 min at room temperature. The grid was then washed with DPBS and fixed with 1% glutaraldehyde (Sigma-Aldrich). TEM images were captured with a JEOL JEM-2000EXII microscope (JEOL, LTD). For NTA, exosomes were diluted to 10^6^ to 10^9^ particles/ml to optimize particle concentration in a view of field for analysis of exosome size distribution using a NanoSight instrument (NS300, Malvern) equipped with a CMOS camera and a 488-nm blue laser.

### Fluorescent labeling and transfer of exosomes

A PKH26 Red Fluorescent Cell Linker Kit (Sigma-Aldrich) was utilized for cell tracing. The exosome pellets were resuspended in 300 μl of Buffer Diluent C (B.C) to make 2× exosome solution. Then, 4 μl of PKH26 dye was added to 1 ml of B.C to make a 2× dye solution. Equal volumes of dye solution were added immediately to exosome suspensions, which were then incubated for 5 min at room temperature and mixed gently during staining. Then, 10% BSA prepared in DPBS (600 μl) was added to the exosome-dye mixture to quench staining for 1 min at room temperature, and 1% BSA was added to reach a final volume of 3 ml. Next, 20 μg/ml of PKH26-labeled or unlabeled exosomes was added onto seeded recipient cells overnight before imaging with a Zeiss LSM880 laser scanning confocal system (Carl Zeiss). Images were processed with ZEN 2009 Light Edition software (Carl Zeiss).

### Immunofluorescent assay

Cells were resuspended in 50 μl of FBS (Gibco) and spread on coated slides to air dry. Cells were then fixed with 4% paraformaldehyde (Sigma-Aldrich) for 20 min at room temperature, washed with DPBS, permeabilized with 0.5% Triton X-100/PBS for 3 min at room temperature, and blocked in 5 mg/ml BSA (Sigma-Aldrich) for 30 min before being probed with a FLAG.Tag (1:200, F1804, Sigma-Aldrich) or p65 (1:200, 10745-1-AP, Proteintech) primary antibody followed by an anti-mouse-fluorescein secondary antibody (1:200, F-2761, Invitrogen) or an anti-rabbit Alexa Fluor-488 (1:200, 21206, Invitrogen). Fluorescence images were visualized with a Zeiss LSM880 laser scanning confocal system (Carl Zeiss). Images were processed using ZEN 2009 Light Edition software (Carl Zeiss).

### RNA sequencing and bioinformatics analysis

Total RNA in neutrophils was extracted with TRIzol (Invitrogen) for RNA sequencing. A TruSeq standard mRNA sample preparation kit (Illumina) was employed for library preparation, and sequencing was performed with a HiSeq 2500 system (Illumina). CLC Genomics Workbench v8.0 software (Qiagen) was employed for trimming reads with a Phred score < 20 and analyzing sequencing data. All sequencing reads were calculated to obtain an RPKM (reads per kilobase of exon model per million mapped reads) value to establish expression profiles. For exosomal RNA sequencing, exosomal RNA was extracted using a miRNeasy mini kit (Qiagen) for library preparation with a TruSeq Small RNA Sample Preparation kit (Illumina). Sequencing reads were run on a NextSeq 500 system (Illumina). Raw reads with a Phred score > 20 were filtered out to clip the 3′ adapter sequence and discard reads shorter than 18 nucleotides using Trimmomatics [20]. For exploring exosomal RNA context, we utilized the framework of ncPRO-seq [21] implemented with small noncoding RNA (sncRNA) features from RepeatMasker (http://www.repeatmasker.org) and Rfam [22]. The expression level of sncRNA was normalized by feature-shared read counts to solve multiple mapping issues. The sequence reads of exosome-stimulated profile of neutrophils and exosomal RNAs were deposited at GSE101951 and GSE101950, respectively. Gene Set Enrichment Analysis (GSEA) (http://software.broadinstitute.org/gsea/index.jsp) was used to assess the degree of association between defined signatures and profiles downloaded from a GSE43254 dataset using the JAVA program. The dataset was collapsed into gene symbol, and gene set was employed for permutation. The gene ontology and connectivity network were established with Ingenuity Pathway Analysis (IPA) software (Ingenuity Systems, Redwood City, CA, USA). The functional categories with *z* score ≥ 2 and *P* values < 0.05 were considered enriched biological features. The normalized RNA sequencing reads of CRC patients in the GDC TCGA COAD dataset was downloaded from UCSC Xena (https://xena.ucsc.edu/), and the median expression of SNAI and IL8 was set for patient stratification.

### Real-time quantitative PCR (RT-qPCR) validation

qPCR was performed using a StepOne-Plus real-time PCR system (Applied Biosystems Inc.). Cellular gene and cellular miRNA expression were normalized to *Gapdh* and *U6*, respectively. The expression of exosomal miRNA was normalized to *U6*. The primers used are indicated here: primer for reverse transcription of miR-146a-5p, GTC GTA TCC AGT GCA GGG TCC GAG GTA TTC GCA CTG GAT ACG ACA ACC CA; primers for qPCR of *miR-146a-5p*, (forward primer) GGC GAT GAG AAC TGA ATT CCA and (reverse primer) TG CAG GGT CCG AGG T; *Numb*, (forward primer) CAA CAC TGC TCC ATC CCC AT and (reverse primer) AAT CCC CGG AAA GAG CCT TG); *CD44*, (forward primer) TGC CTC AAC TGT GCA CTC AA and (reverse primer) GTT CTG GGC TTC TTG CCT CT; *Ascl2*, (forward primer) CTA CTC GTC GGA GGA AAG CA and (reverse primer) ACT AGA CAG CAT GGG TAA GGC; *Olfm4*, (forward primer) TAC GAG TTC TGC GGA GGG AT and (reverse primer) TTG CTT TCC ACT CGT GCT CC; *Cdx2*, (forward primer) CTTTGTCAGTCCTCCGCAGT and (reverse primer) CGTAGCCATTCCAGTCCTCG; *Bmp4*, (forward primer) GCT AGG TGA GTT CGG CAT CC and (reverse primer) GAG AAT CCC ATC AGG GAC GG; *Il-1b*, (forward primer) GGT CAA AGG TTT GGA AGC AG and (reverse primer) TGT GAA ATG CCA CCT TTT GA; *Gapdh*, (forward primer) GTG CAG TGC CAG CCT CGT CC and (reverse primer) GCC ACT GCA AAT GGC AGC CC; *U6*, (forward primer) CTC GCT TCG GCA GCA C and (reverse primer) AAC GCT TCA CGA ATT TGC G.

### Flow cytometry analysis and cell sorting

An RBC-depleted, single-cell suspension was prepared in blocking solution (2 mM EDTA, 1% (*w*/*v*) BSA in DPBS) and hybridized with the following fluorescence-conjugated primary antibodies: anti-CD11b-PE (1:25, 130-091-240, Miltenyi Biotech), anti-GR1-APC (1:25, 130-102-385, Miltenyi Biotech), anti-CD11b-APC (1:25, 130-091-241, Miltenyi Biotech), anti-Ly6G-PE (1:25, 130-102-392, Miltenyi Biotech), anti-Ly6C-FITC (1:25, 130-102-295, Miltenyi Biotech), anti-Ly6G-PerCp-Vio700 (1:25, 130-103-861, Miltenyi Biotech), anti-Ly6A/E(ScaI)-PE (1:25, 561076, Becton-Dickinson), anti-CD1147(c-Kit)-APC (1:50x, 561074, Becton-Dickinson), anti-CD34-FITC (1:25, 560238, BD), anti-CD16/32-PE.Cy7 (1:50, 25-0161, Affymetrix eBioscience), anti-Lin-PerCp.Cy5.5 cocktail (1:50, 561317, Becton-Dickinson), anti-CD44-APC (1:50, 103011, Biolegend), anti-Prominin-1-PE (1:50,130-102-834, Miltenyi Biotech), anti-Lgr5-VioBright.FITC (1:50, 130-111-393, Miltenyi Biotech), IκBα (1:200, 10745-1-AP, Proteintech), and Alexa Fluor-488 (1:200, 21206, Invitrogen). For intracellular staining of IκBα, a fixation and permeabilization kit was applied (BD cytofix/cytoperm, Becton-Dickinson). Cell debris was excluded from analysis based on scatter signals, and fluorescent compensation was adjusted when cells were stained with multiple fluorescence-labeled antibodies. The positive populations were defined by comparison to isotype control or a nonstaining group. Only cells with purity greater than 80% upon sorting were subjected to further analysis. A FACS Calibur flow cytometer (Becton-Dickinson) was employed for data acquisition and a FACSAria system (Becton-Dickinson) was applied for cell sorting. CellQuest (Becton-Dickinson) and FlowJo software (TreeStar) were used for analysis.

### Reactive oxygen species (ROS) detection

For detecting ROS levels, a DCFDA-cellular ROS detection kit (Abcam) was used. Briefly, cells were incubated with 20 μM DCFDA for 1 h at 37 °C, and administration of 500 nM TBHP (2 h at 37 °C) was used as a positive control. All cells were collected without further washes as suggested by the manufacture’s protocol for flow cytometry analysis.

### Apoptosis assay

The apoptotic status of cells was evaluated using an Alexa Fluor@488 Annexin V/Dead cell apoptosis kit (Invitrogen), a FACS Calibur flow cytometer (Becton-Dickinson), and CellQuest (Becton-Dickinson) software.

### Phagocytosis assay

The uptake of pHrodoRed E. coli Bioparticles (Invitrogen) was used to assess phagocytosis. The 2× diluent E. coli bioparticle stock (2 mg/ml) was prepared in HBSS (pH = 7.4, Gibco) and vortexed well to disperse the particles. Then, 3 × 10^4^ cells were resuspended in 100 μl of HBSS, and an equal volume of E. coli bioparticles was added to the cell mixture for 1 h at 37 °C and 4 °C (a negative control). The cells were then washed with HBSS and resuspended in cold DPBS (Gibco) for flow cytometry analysis.

### In vitro Transwell cell migration

Cell migration ability was evaluated using a 3-μm filter membrane containing an upper chamber (Corning). Then, 1 × 10^6^ cells were suspended in 100 μl of basal medium and added to the upper chamber of the device and lower chamber containing 600 μl of SDCSC medium plus 5 μg/ml CXCL1 neutralizing antibody (AF-453-NA, R&D Systems Inc.), 5 μg/ml of CXCL2 neutralizing antibody (AF-452-NA, R&D System Inc.), or 10 μg/ml of control IgG antibody (AB-108-C, R&D System Inc.) for 6 h. Cells in the lower chambers were collected by centrifugation and subjected to flow cytometry to determine the cell number.

### T-cell proliferation assay

Splenic CD4 T-cells from Balb/C mice were stained with an anti-CD 4-FITC antibody (1:25, 130-102-541, Miltenyi Biotech). CD3/CD28 T-cell activator Dynabeads (Gibco) were suspended and washed with blocking buffer (2 mM EDTA, 0.1% BSA in PBS) on a magnetic stand. Then, 8 × 10^4^ CD4 T cells were seeded per well in a 96-well plate in neutrophil conditioned medium, and 2 μl of prewashed T cell activator Dynabeads beads were added per well for 72 h. The proliferation of T cells was measured by cell counting.

### Drug resistance and MTT assay

Briefly, 1 × 10^4^ cells per well were seeded in a 96-well plate in complete DMEM and incubated overnight before treatment with 5-FU (Haupt Pharma Wolfratshausen Gmbh) for 48 h or Etoposide (Fresenius Kabi Oncology Ltd) for 72 h. After drug treatment, the medium was discarded, and MTT reagent (Sigma-Aldrich) was added to cells for 1 h at 37 °C. The mitochondrial MTT crystals were dissolved with DMSO (J.T Baker), and then, the absorbance was read with a microplate reader (Spectramax 250, Molecular Devices Corp).

### IL-1β ELISA

Conditioned medium was collected and twofold dilutions were made to detect the mouse IL-1β concentration using an ELISA (eBioscience) in accordance with the manufacture’s protocol, and the results were read with a microplate reader (Molecular Devices Corp).

### Plasmids and synthetic oligonucleotides

The CD81 fragment of mPA-GFP-CD81-10 (Plasmid #57124, Addgene, Cambridge, MA, USA) was employed to generate pCDH-FLAG-CD81. The CD81 fragment was first amplified from an Addgene clone (#57124) using the indicated primers (forward primer: ATCGGCG GCC GC A GGA GTG GAG GGC TGC ACC A; reverse primer: ATCGCCC GGG TCA GTA CAC GGA GCT GTT CC) and ligated into a pFLAG-CMV2 vector at the NotI/SmaI sites to generate a FLAG-tagged CD81 insert. The FLAG-CD81 insert was then amplified using subcloning primers (forward primer: ATCGGAA TTC ATG GAC TAC AAA GAC GAT GAC G; reverse primer: ATCGGGA TCC TCA GTA CAC GGA GCT GTT CCG) and ligated into a pCDH-puro vector (CD510B-1, System Biosciences) at the EcoRI/ BamHI sites to generate pCDH-FLAG-CD81. Flanking sequence: ATCG (underlined). One additional in frame nucleotide: A (underlined). All clones were verified by direct sequencing. The micrOFF® miRNA antagomir against miRNA-146a (miR30000449-1-10) and micrOFF® antagomir Negative Control (miR03201-1-10) were purchased from RiboBio (RiboBio Co., Ltd).

### Immunoblotting

Protein extracts were quantified using a Pierce BCA Protein Assay Kit (Thermo Fisher Scientific) according to the manufacturer’s protocol. The transferred membrane was blocked and hybridized with the following antibodies in 5% BSA (Sigma-Aldrich) overnight at 4 °C: anti-ALIX (1:1000, 2171S, Cell Signaling), anti-TSG101 (1:1000, EXOAB-TSG101-1, System Biosciences), anti-CD81 (1:1000, GTX101766, Genetex), anti-Numb (1:1000, ab14140, Abcam), anti-β-catenin (1:1000, 610154, Becton-Dickinson), anti-CXCL1 (1:1000, AF-453-NA, R&D Systems Inc.), anti-CXCL2 (1:1000, AF-452-NA, R&D System Inc.), anti-FLAG tag (1:1000, F1804, Sigma-Aldrich), and anti-β-actin (1:5000, A5441, Sigma-Aldrich). The corresponding secondary antibodies were used for hybridization at room temperature for 1 h: bovine anti-rabbit IgG-HRP (1:3000, sc-2370, Santa Cruz Biotechnology), chicken anti-goat IgG-HRP (Catalog sc-2953, Santa Cruz Biotechnology), and chicken anti-mouse IgG-HRP (1:5000, sc-2954, Santa Cruz Biotechnology). Immunoblots was visualized with a chemiluminescence detection system (ImageQuant LAS 4000, GE Healthcare Bio-Sciences).

### Animal experiments

The animal study was approved by the Committee on the Ethics of Animal Experiments of Taipei Medical University (Permit Number. LAC-2015-0176). Balb/C mice aged 5 to 8 weeks old were purchased from National Laboratory Animal Center at Taiwan for tumorigenicity, immunocyte isolation, and tail vein injection. (A). Tumorigenicity was evaluated by using orthotropic transplantation and subcutaneous tumor injection in a syngeneic tumor model. For orthotropic transplantation, CRC cells were suspended in 10 μl basal DMEM and injected into the subcapsular region of the cecum of anesthetized mice and allowed to proliferate for 4 weeks. For subcutaneous injection, CRC cells were suspended in 50 μl of DMEM and mixed with an equal volume of Matrigel (Becton-Dickinson) prior to injection. Then, 100 μg per injection of *InVivo*Plus anti-mouse Ly6G antibody (clone 1A8, BioXcell) or corresponding isotype control (clone 2A3, BioXcell) was administered to mice via intraperitoneal (i.p.) injection every 4 days for 30 days. (B). For isolation of tumor-infiltrated myeloid cells, tumors were cut into 2-mm^3^ fragments and digested with 1.5 mg/ml collagenase IV (Sigma) for 1 h at 37 °C. Tissue fragments were dissociated by pipetting for 15 min to release single cells. The tumor-infiltrated immune cells were enriched by density gradient centrifugation and collected at the 44–67% interface of Percoll (GE Healthcare Life Science). (C). To identify cells engulfing exogenous exosomes in the bone marrow, exosomes derived from FLAG-tagged CD81-expressing CT26-SDCSCs were collected, and 15 μg of exosomes were diluted in 100 μl of DPBS for one injection via the tail vein. To validate the effects of CT26-SDCSC exosomes on the number of neutrophils and monocytes, CT26-SDCSC exosomes were injected every 3 days (3 μg of exosomes in 100 μl of DPBS for one tail vein injection), and a total of 45 μg exosomes were administered. Host cells were either isolated from femurs or the spleen, and red blood cells (RBCs) were lysed in hypotonic solution. Cells were then suspended in blocking solution (2 mM EDTA, 1% BSA) for antibody staining and cell sorting. For complete blood cell counts (CBCs), whole blood was collected in anti-coagulant EDTA tubes (Becton-Dickinson) by cardiac puncture and analyzed using an automated hematology analyzer (Sysmex XT-1800iv, Sysmex Canada Inc.) according to the manufacturer’s protocol.

### Statistical analysis

Independent sample *t* tests were performed to compare continuous variation between two groups, and a *χ*^2^ test was applied for comparison of dichotomous variables. *P* values < 0.05 were considered significant. The data are presented as the mean ± S.D. or as described in the figure legends. For animal studies, no statistical method was used to predetermine sample size.

## Results

### Expansion and characterization of murine CRCSCs

**Fig. 1:**
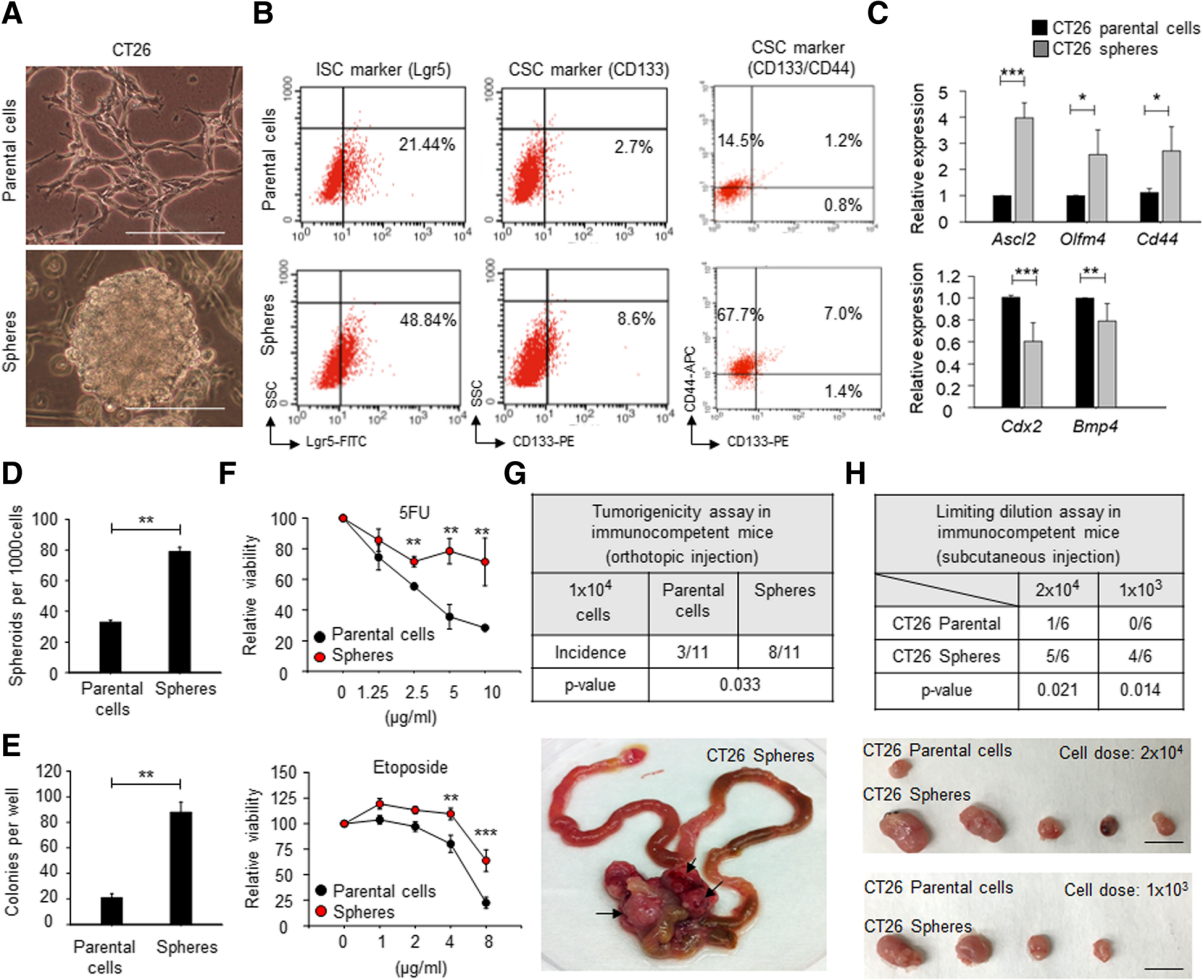
The expanded SDCSCs show enhanced stem cell property, chemoresistance, and tumor-initiating capacity. **a** Representative images of CT26 parental cells and SDCSCs. Scale bar = 100 μm. **b** Flow cytometry showing expression of Lgr5 (left panels) and CD133 (middle panels) and CD133(+)/CD44(+) (right panels) in CRC cells. The percentage of immunoreactivity positive cells are indicated. **c** RT-qPCR validation of expression of stemness genes (upper panel) and differentiation genes (lower panel). *P < .05, **P < .01, ***P < .001. **d** A histogram for showing sphere-forming capacity of CRC cells. **P < .01. **e** A histogram showing clonogenicity of CRC cells. Data represent mean ± S.D. **P < .01. **f** The scatter plots demonstrating viability of CRC cells under 5-FU (upper panel) and etoposide (lower panel) treatment. **P < .01, ***P < .001. **g** The orthotopic cecum tumorigenicity. Upper: a table summarizing tumor incidence. Lower: a representative image for successful grown tumors from CT26-SDCSCs. Arrow, growing tumors. **h** Tumor-initiating potentials. Upper: a table summarizing the tumorigenicity. Lower: the representative images for tumor grown. Scale bar = 1 cm

We initiated this study by expanding CRCSCs from a murine CRC cell line, CT26, using a serum-free, spheroid cultivation method to prepare cells for subsequent in vitro and syngeneic animal experiments because enriched tumor spheres retain their original genetic features and phenotypes in primary tumors [23]. The resultant CT26 colonospheres (Fig. 1a, bottom panel) showed increased populations expressing the intestinal stem cell (ISC) marker, Lgr5 (Fig. 1b, left panels), and CSC marker, CD133 (Fig. 1b, middle panels), as well as CD133/CD44 double positive cells (Fig. 1b, right panels). The CT26 colonospheres also showed enhanced expression of stemness genes (*Ascl2*, *Olfm4*, and *Cd44*) and decreased expression of differentiation genes (*Cdx2* and *Bmp4*) (Fig. 1c). Enhanced sphere-forming ability (Fig. 1d), clonogenicity (Fig. 1e), chemoresistance (Fig. 1f), orthotropic tumorigenicity (Fig. 1g), and tumor-initiating capacity (Fig. 1h) were observed in CT26 spheres. Here, the enriched CT26 colonospheres harbored critical features of CSCs and were defined as sphere-derived cancer stem cells (SDCSCs, hereafter).

### Systemically increased neutrophils in mice bearing CRCSC-derived tumors

**Fig. 2:**
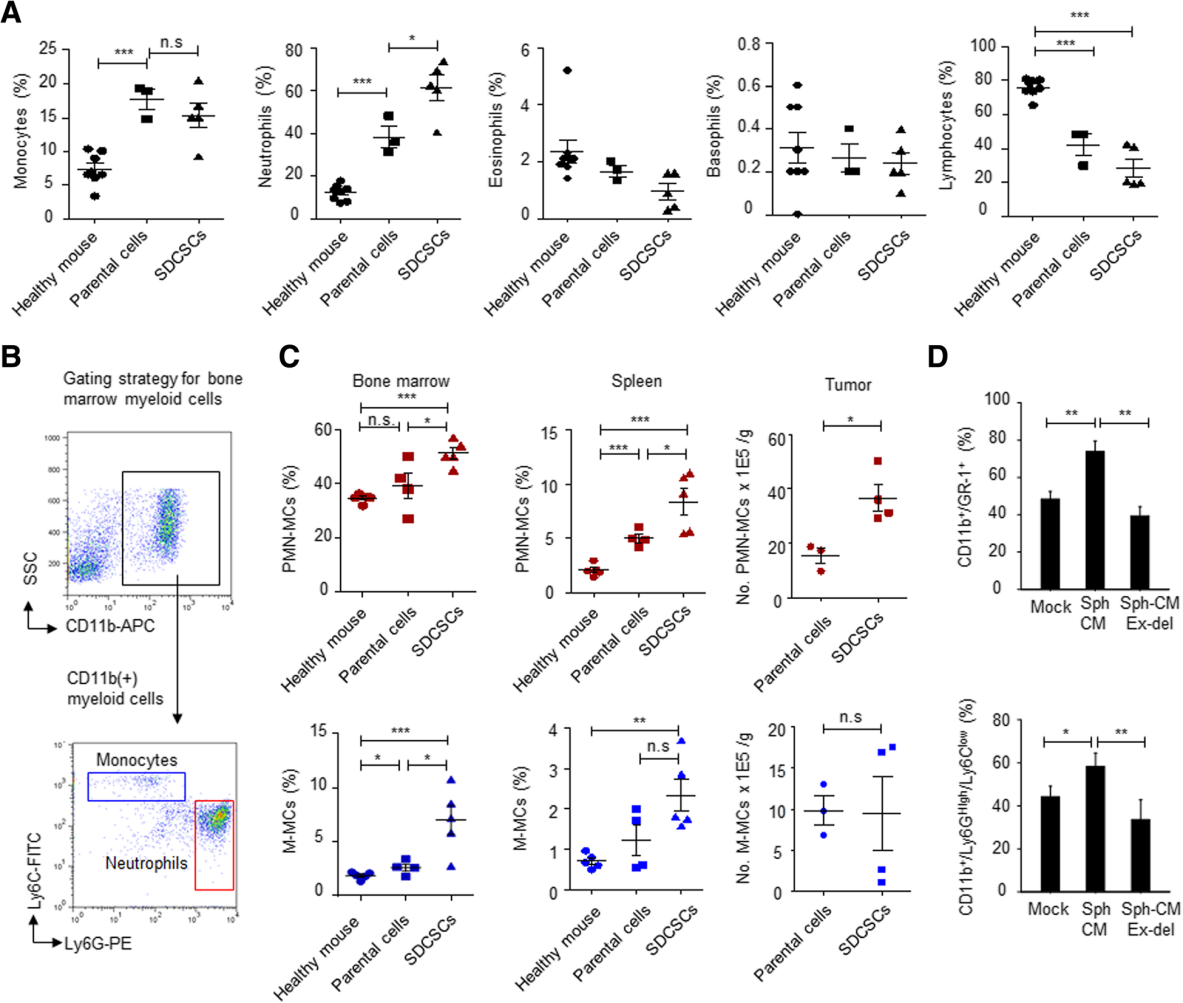
Systemic expansion of neutrophils in mice bearing SDCSC-derived tumors. **a** The percentage of circulating monocytes, neutrophils, eosinophils, basophils, and lymphocytes from indicated healthy mice (*N* = 8), mice bearing CT26 parental cell-derived tumor (*N* = 3), and mice bearing CT26-SDCSC-derived tumor (*N* = 5). Data represent the mean ± SEM. *P < .05, ***P < .001. **b** Gating strategy for detecting neutrophils and monocytes. **c** Percentage of neutrophils (PMN-MCs, polymorphonuclear myeloid cells) and monocytes (M-MCs, mononuclear myeloid cells) in bone marrows (left panels), spleens (middle panels), and primary tumors (right panels) harvested from healthy mice (*N* = 5), mice bearing parental cell-derived tumor (*N* = 4 for bone marrow and spleen groups, *N* = 3 for tumor group), and mice bearing SDCSC-derived tumor (*N* = 5 for bone marrow and spleen groups, *N* = 4 for tumor group). The data represent mean ± SEM. *P < .05, **P < .01, ***P < .001. **d** Percentage of CD11b^+^/Gr-1^+^ neutrophils (upper panel) and CD11b^+^/Ly6G^HIgh^/Ly6C^low-neg^ neutrophils in ex-vivo cultured bone marrow cells. Indicated medium was mixed with complete RPMI medium at 1:1 ratio for 72 h. Mock, complete DMEM medium; SCM, stem cell medium; Sph-CM, condition medium from SDCSCs; Sph-CM ex-del, exosome-removed condition medium of SDCSCs. *P < .05, **P < .01, ***P < .001

To explore CSC-host interaction, we utilized a Balb/C syngeneic tumor model to examine the immune cell composition in CRCSC tumor-bearing mice. Using complete blood cell counts (CBCs), an increase in monocytes, neutrophils, and a reduction in lymphocytes were noted in mice bearing syngeneic tumors derived from both CT26 parental cells and SDCSCs compared with normal mice. A significant increase in circulating neutrophils was observed in mice bearing SDCSC-derived tumors compared with those bearing parental CT26-derived tumors (Fig. 2a). The systemic distribution of host myeloid cells in tumor-bearing mice was confirmed by examining surface markers of neutrophils (CD11b^+^/Ly6G^High^/Ly6C^Low^) and monocytes (CD11b^+^/Ly6C^High^/Ly6G^Low^) (Fig. 2b). We found an increased number of neutrophils in the bone marrow, spleens, and localized tumors from mice bearing CT26-SDCSC-derived tumors compared with mice with parental CT26-derived tumors. Although an increased number of monocytes was found in the bone marrow and spleens of mice bearing CT26-SDCSC-derived tumors, nevertheless, the percentage of monocytes in both the spleens and tumors from SDCSC-tumor bearing mice was not increased over that in spleens and tumors from mice bearing parental-derived tumors (Fig. 2c), indicating potential roles of neutrophils in the tumorigenesis of CRCSCs. Exosomes were discovered in rat reticulocytes in 1983 [24] and have becomes relevant to many fields including cancer vaccines [25], drug delivery [26], and cell-cell communications [27]. As exosomes and other secretory proteins (i.e., cytokines and growth factors) are both crucial secreted cellular components, we then investigated the both components in CSC-neutrophil interaction. By culturing total bone marrow cells in conditioned medium from CT26-SDCSCs (Sph-CM) and exosome-depleted CM (Sph-CM Ex-del), the SDCSC-released exosomes but no other secreted components were found to be required for expansion of CD11b^+^/Gr-1^+^ and CD11b^+^/Ly6G^High^/Ly6C^Low^ neutrophils (Fig. 2d).

### Bone marrow transportation of murine CRCSC exosomes

**Fig. 3:**
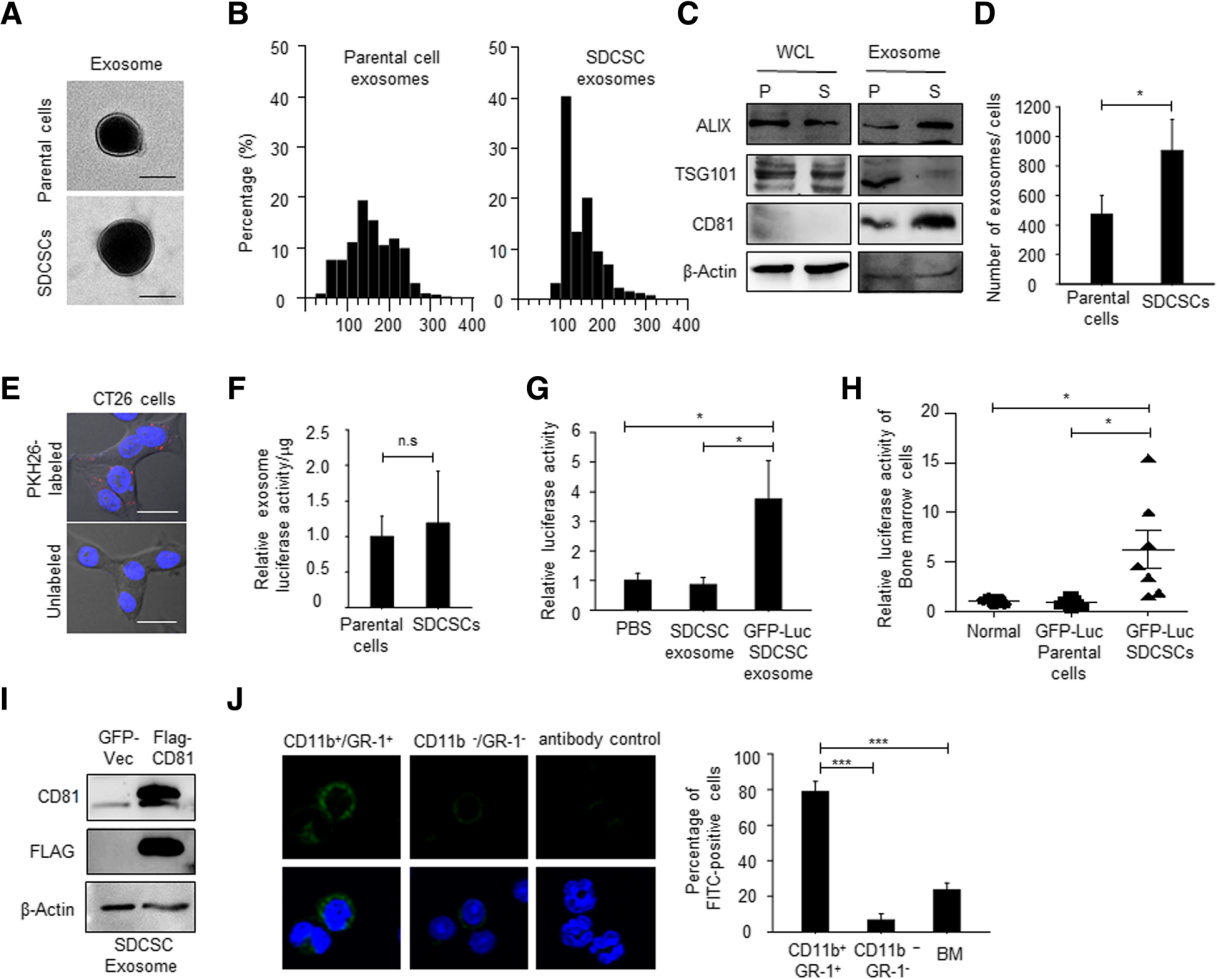
SDCSC exosomes are transported to bone marrows and increase neutrophil population in bone marrow. **a** Representative images of exosomes captured with TEM. Scale = 100 nm. **b** The size distribution of exosomes by NTA. **c** Western blots of exosome markers in cells. P, parental cells; S, SDCSCs; WCL, whole cell lysate. **d** Histograms showing relative exosome secretion in CRC cells. *P < .05. **e** The representative images for transferring of PKH26-labeled exosomes. Scale bar = 10 μm. **f** The relative luciferase activity of purified exosomes. **g** The relative luciferase activity of CT26 cells receiving CT26-SDCSC-released exosomes. GFP-Luc-SDCSC exosome, exosomes from GFP-luciferase-expressing SDCSCs. *P < .05. **h** The relative luciferase activity in bone marrow cells of normal mice (*N* = 5), mice with tumors grown from GFP-Luc parental cells (*N* = 7), and mice with GFP-Luc SDCSC-derived tumors (*N* = 7). The data represent mean ± SEM. *P < .05. **i** Western blot of CD81 and FLAG from 10 μg of exosome proteins purified from ectopically FLAG-CD81-expressing SDCSCs and GFP-expressing control cells. **j** Left: representative images of FLAG-FITC signals in cells. Right: quantification of FLAG-FITC positive cells. ***P < .001

Given the potential involvement of neutrophils in CRCSC-related malignancy, we collected tumor exosomes from CT26-SDCSCs to investigate the effects of CRCSC exosomes on neutrophils. It was found that tumor exosomes derived from CT26 parental cells and SDCSCs showed a membranous morphology (Fig. 3a), with diameters ranging from 50 to 200 nm based on TEM (Fig. 3b). The expression of exosome markers, including CD81, ALIX, and TSG10, was detectable in both types of tumor exosomes (Fig. 3c), and an increased exosome secretion was observed in CT26-SDCSCs using NTA (Fig. 3d). The successful transfer of fluorescent PKH26-labeled SDCSC exosomes to parental CT26 cells further indicated their bioactivity as cargos for cell-cell communication (Fig. 3e).

The bone marrow is widely known as a reservoir for neutrophils [28]. In an attempt to investigate the bone marrow transportation of CRCSC exosomes in vivo, we utilized two models for tracking exosomes. First, luciferase-expressing CRC cells were generated, and released tumor exosomes were collected to examine luciferase activity. We found that the exosomal luciferase activity was comparable in parental and SDCSC-derived exosomes (Fig. 3f) and that SDCSC-exosomal luciferase could be transferred to recipient CT26 parental cells, suggesting the feasibility of utilizing luciferase-carrying exosomes for in vivo tracking (Fig. 3g). An elevated luciferase activity in bone marrow cells was detected in mice bearing SDCSC-derived tumors expressing GFP-Luciferase (Fig. 3h). Second, when we administered purified SDCSC exosomes ectopically expressing FLAG-tagged CD81 (Fig. 3i) via a tail vein injection into tumor-free mice, CD11b^+^/Gr-1^+^ neutrophils were the predominant group engulfing exogenous tumor exosomes in the bone marrow (Fig. 3j). Overall, increased secretion of CRCSC exosomes may contribute, at least in part, to their bone marrow transportation.

### CRCSC exosomes prolong viability and promote a pro-tumoral phenotype in bone marrow-derived neutrophils

**Fig. 4:**
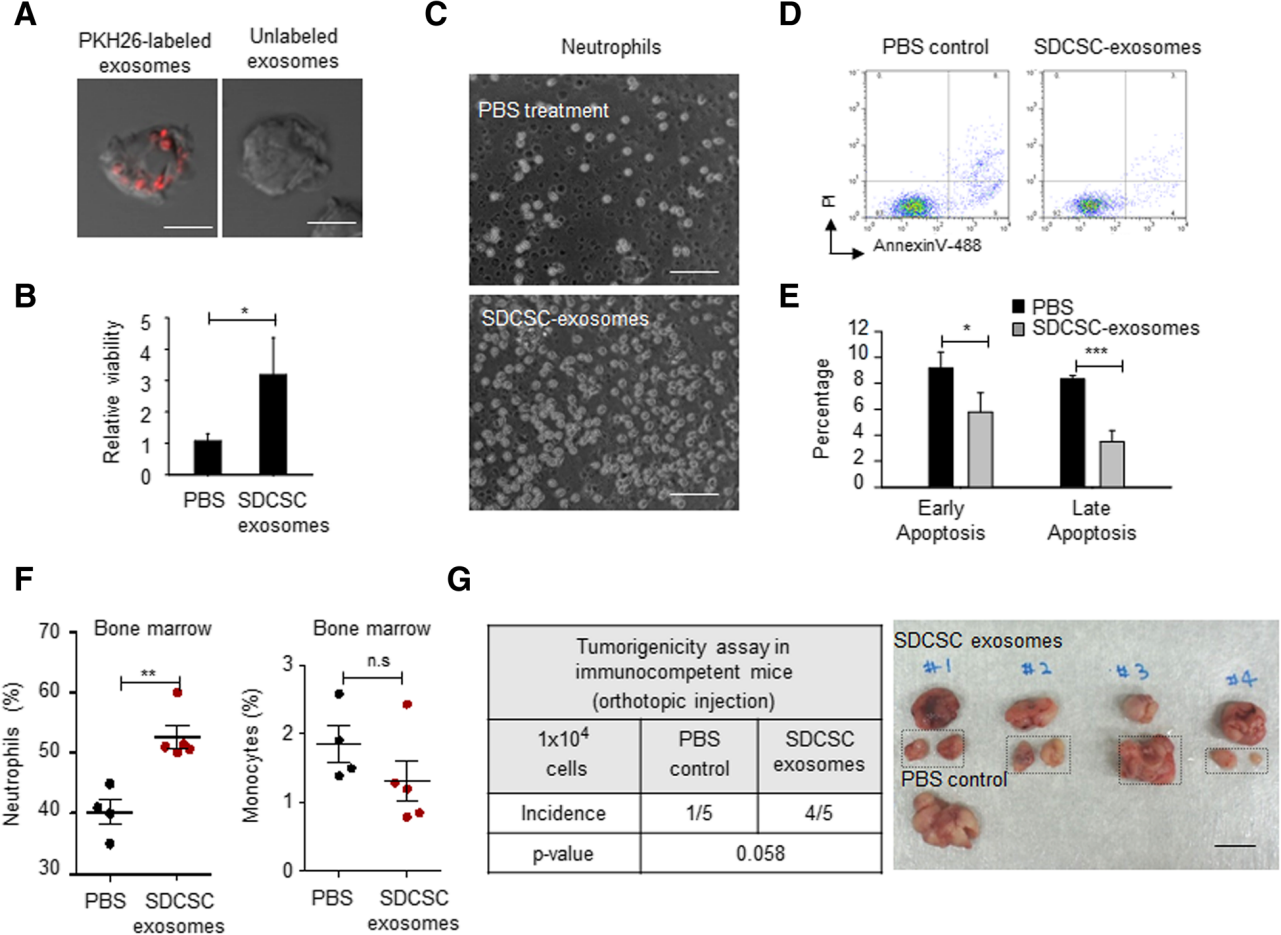
The SDCSC exosomes prolong viability of neutrophils and introduce tumor-promoting feature to bone marrow-derived neutrophils. **a** Representative images of internalized PKH26-labeled SDCSC exosomes in neutrophils. Scale bar = 5 μm. **b** Relative viability of neutrophils treated with PBS or 20 μg/ml of SDCSC exosomes for 96 h. *P < .05. **c** Representative images of neutrophils upon treatment at day 3. Scale bar = 25 μm. **d** Representative flow cytometry results showing early and late apoptotic neutrophils. **e** A histogram showing the percentage of cells in early apoptosis (Annexin V: positive/ PI: negative cells) and late apoptosis (Annexin V: positive/PI: positive cells). PI, propidium iodide. *P < .05, ***P < .001. **f** Percentage of neutrophils (left panel) and monocytes (right panel) in bone marrows of mice injected with CT26-SDCSC exosomes or PBS. A total of 45 μg exosomes were injected through tail vein. PBS, mice receiving PBS injection (*N* = 4); SDCSC exosomes, mice-receiving SDCSC exosome injection (*N* = 5). The data represent mean ± SEM. **P < .01. **g** The orthotopic tumorigenicity assay. Left: a table summarizing the tumor incidence. Right: a representative images of grown tumors. The rectangles indicate tumors harvested from peritoneal cavity. Scale bar = 1 cm

Next, we elucidated the impact of CRCSC exosomes on bone marrow-derived neutrophils. First, the uptake of fluorescent PKH-labeled CT26-SDCSC exosomes was confirmed in neutrophils sorted from tumor-free mice (Fig. 4a). The neutrophils retained their phagocytosis function (Additional file 1: Figure S1A), ability to generate reactive oxygen species (ROS) (Additional file 1: Figure S1B), and expression of CXCR2 (a key mediator for neutrophil mobilization) (Additional file 1: Figure S1C), when treated with CT26-SDCSC exosomes. Increased viability (Fig. 4b, c) and decreased apoptosis (Fig. 4d, e) were observed in neutrophils upon administration of CT26-SDCSC exosomes. Additionally, an increased number of neutrophils but not monocytes were observed in the bone marrow when CT26-SDCSC exosomes were repeatedly administered to tumor-free mice via a tail vein injection (Fig. 4f). However, the populations of lineage marker(−)ScaI(+)Kit(+) hematopoietic stem cells (LSK-HSCs), granulocyte-macrophage progenitors (GMPs), common myeloid progenitors (CMPs), and megakaryocyte-erythroid progenitors (MEPs) were not altered in mice receiving CT26-SDCSC exosome injections (Additional file 2: Figure S2A-B), indicating neutrophil expansion may be independent of proliferation of myeloid progenitors. The expanded neutrophils in the bone marrow were not proportional to the neutrophil numbers in the spleen upon SDCSC exosome injection (Additional file 2: Figure S2C). Additionally, coinjection of SDCSC exosome-primed neutrophils with CT26 parental cells enhanced tumorigenesis of CT26 cells and their peritoneal spreading in vivo (Fig. 4g). Based on the above results, CRCSC exosomes confer a growth advantage and induce a pro-tumoral phenotype in neutrophils.

### CRCSC-exosomal-RNA-induced interleukin-1β expression sustains survival of neutrophils

**Fig. 5:**
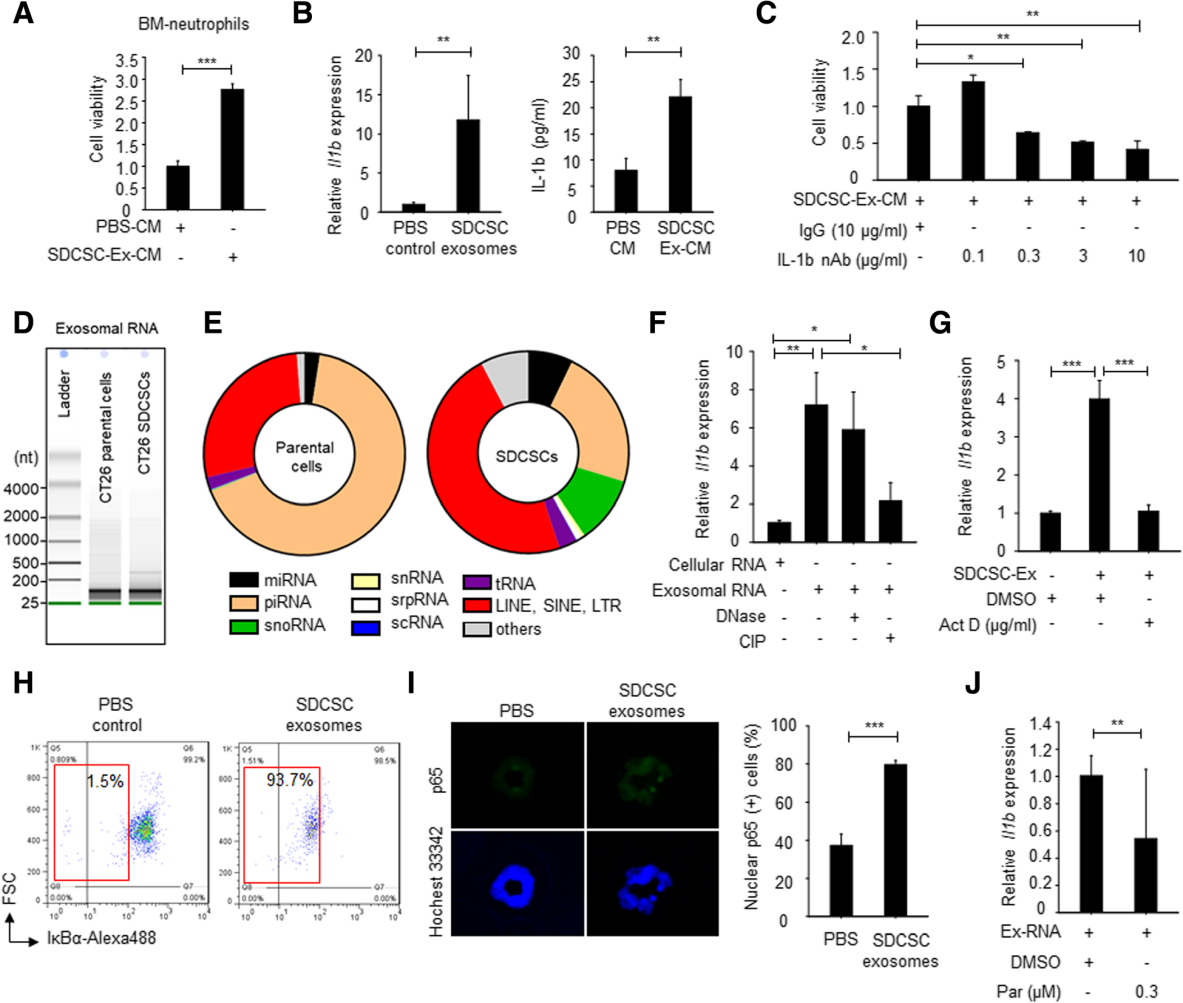
Systemic biology analysis identifies expression of exosomal RNAs-induced interleukin-1β is required for neutrophil survival. **a** Viability of neutrophils treated with different condition medium of educated-neutrophils. PBS-CM, conditional medium from PBS-treated neutrophil; SDCSC-Ex-CM, condition medium from SDCSC exosome-treated neutrophils. ***P < .001. **b** Validation of IL-1β expression using RT-qPCR (left panel) and ELISA (right panel) under indicated conditions. **P < .01. **c** A histogram showing neutrophil viability under indicated conditions. IgG, normal IgG control (10 μg/ml); Il-1b nAb, neutralizing IL-1β antibody. *P < .05, **P < .01. **d** Representative images for showing size distribution of exosomal RNA by Agilent Bioanalyzer. nt, nucleotide. **e** Pie charts showing exosomal RNA context of parental cells and SDCSCs. miRNA, microRNA; piRNA, Piwi-interacting RNA; snoRNA, small nucleolar RNA; snRNA, small nuclear RNA; srpRNA, signal recognition particle RNA; scRNA, small cytoplasmic RNA; tRNA, transfer RNA; LINE, long interspersed nuclear elements; SINE, short interspersed nuclear elements; LTR, long terminal repeat. **f** RT-qPCR examining *Il1b* expression in neutrophils upon transfection. Cellular and exosomal RNAs were extracted from CT26-SDCSCs. CIP, calf intestinal phosphatase. *P < .05, **P < .01. **g** RT-qPCR examining *Il1b* expression in neutrophils. Act D, actinomycin D (0.3 μg/ml). ***P < .001. **h** Flow cytometry results for showing expression of IκBα. **i** Left: Representative images illustrating nuclear p65 expression. Right: a histogram for showing percentage of nuclear p65(+) cells. ***P < .001. **j** RT-qPCR showing *Il1b* expression in neutrophils upon blocking NFκB pathway. Exosomal RNA was extracted from CT26-SDCSCs. Parthenolide, a NFκB inhibitor (Par, 0.3 μM). Cells were transfected with 100 ng of exosomal RNAs for 6 h followed by parthenolide or DMSO treatment for a total of 24 h. *P < .05, **P < .01

Because neutrophils are short-lived cells, exploration of the molecular determinants mediating the survival of CRCSC-educated neutrophils is critical. We subjected exosome-stimulated neutrophils and control counterparts to RNA sequencing analysis and bioinformatics analysis. The differential expression profile of SDCSC exosome-stimulated neutrophils was identified (Additional file 3: Table S1). Moreover, the biological features pertaining to infection and inflammatory response, cell growth and cell cycle, metabolism, trafficking, and homing were enriched in CRCSC exosome-trained neutrophils (Additional file 4: Figure S3A). By analyzing canonical pathways, signaling pathways related to EIF2 signaling and pattern recognition receptors (PRRs) with roles in recognition of bacteria and viruses were identified in CRCSC exosome-treated neutrophils (Additional file 4: Figure S3B). The genes involved in the E2F- and PRR-related signaling pathways were then used to establish a connectivity network. Here, interleukin-1β (IL-1β) was found to be the “hub regulator” connecting to PRR-related molecules (Additional file 4: Figure S3C). As exosome-depleted conditioned medium from SDCSC exosome-treated neutrophils enhanced the survival of fresh isolated neutrophils, we centered on the “hub” secreted protein, IL-1β (Fig. 5a). We found that the expression of *Il1b* (Fig. 5b, left) and secretion of Il-1β (Fig. 5b, right) were increased in neutrophils administered CT26-SDCSC exosomes. Importantly, blocking of IL-1β activity with a neutralizing antibody attenuated the survival of neutrophils cultivated in conditioned medium from SDCSC exosome-treated neutrophils (Fig. 5c).

Pattern recognition receptors (PRRs) have been implicated in the innate immunity mediated by neutrophils for pathogen clearance, and the expression of PRR was increased in SDCSC exosome-educated neutrophils (Additional file 4: Figure S3C, labeled in yellow). Therefore, exosome-loaded pathogen-associated molecular pattern (PAMP) molecules may lead to activation of IL-1β through a pattern recognition response. Tumor exosomes carried short RNAs (Fig. 5d), and thus, distinct exosomal RNA types may result in a pattern recognition response. Through exosomal RNA sequencing, a diverse RNA spectra was found in both parental- and SDCSC-derived exosomes, and retrotransposons, including long interspersed nuclear elements (LINEs), short interspersed nuclear elements (SINE), and long terminal repeats (LTRs), were major RNA components in CT26 SDCSC exosomes (Fig. 5e). It is known that transposable elements are transcribed by RNA polymerase III [29] to acquire a 5′-triphosphate end for RIG-1-dependent pattern recognition [30] and NF-κB activation [31]. Hence, we elucidate the roles of SDCSC-exosomal RNAs in IL-1β expression in neutrophils. We found that SDCSC-exosomal RNAs but not cellular RNAs were able to augment IL-1β expression in neutrophils, and such expression was reduced upon removal of 5-phosphates with calf intestinal phosphatase (CIP) (Fig. 5f). Administration of the transcription inhibitor, actinomycin D (Act D), further suppressed IL-1β expression in SDCSC exosome-treated neutrophils (Fig. 5g). These results suggested IL-1β transcript was not transferred by SDCSC exosomes to neutrophils but induced by the 5-phosphates exosomal RNA pattern in neutrophils. Furthermore, SDCSC exosomes promoted the NF-κB activity in neutrophils as indicated by the decreased IκBα (Fig. 5h) and the increased nuclear P65 accumulation (Fig. 5i). Inhibition of NF-κB activity by parthenolide (Par) also decreased exosomal RNA-induced IL-1β expression in neutrophils, suggesting the involvement of NF-κB activation upon exosomal RNA-PRR interaction (Fig. 5j). Our findings indicate that CRCSC exosomes induce IL-1β expression in neutrophils through exosomal tri-phosphate RNA, leading to prolonged neutrophil survival.

### CRCSC exosome-stimulated neutrophils promote tumorigenesis of CRC cells through secretion of IL-1β

**Fig. 6:**
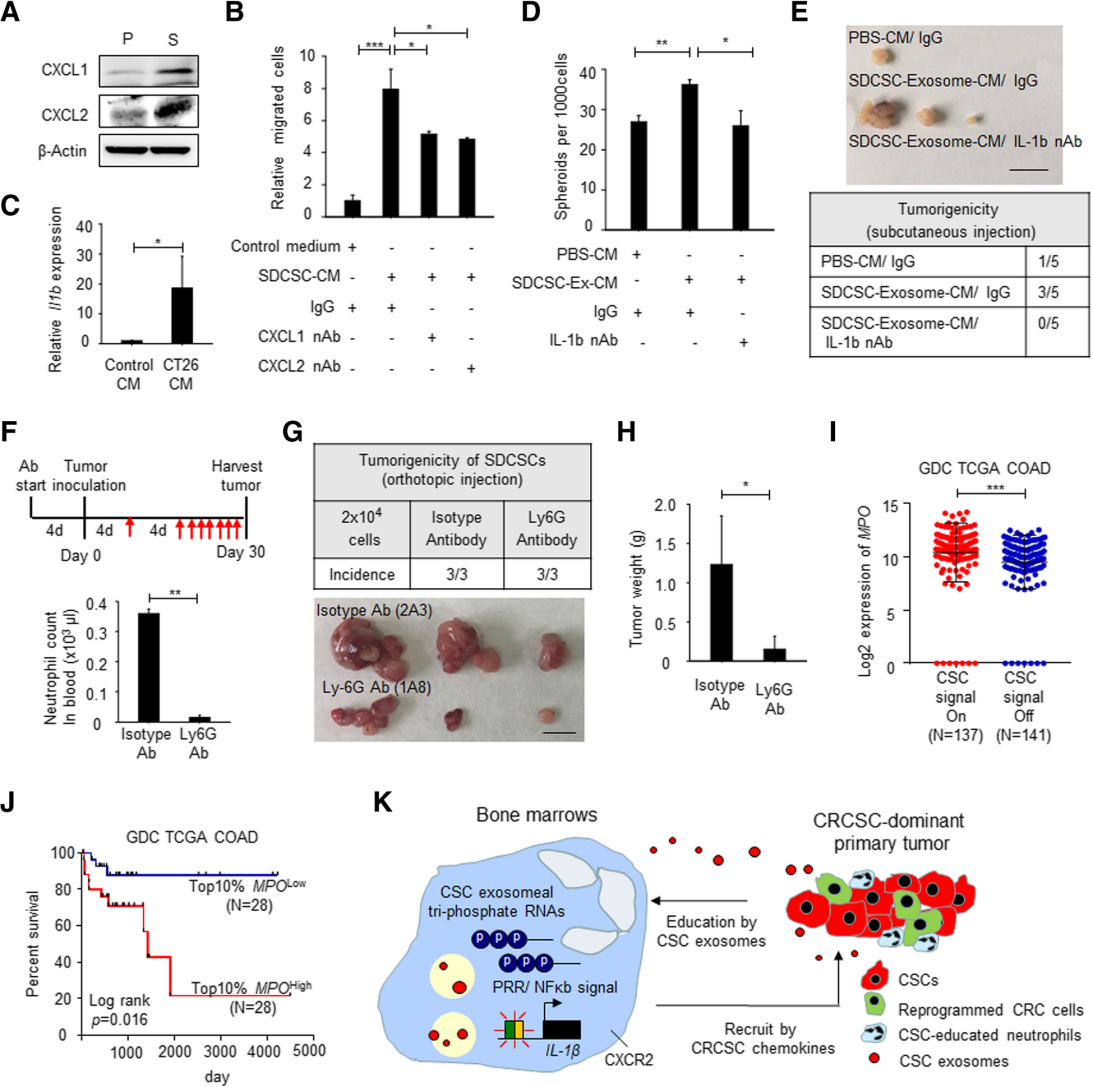
SDCSC-secreted CXCL1 and CXCL2 promote migration of neutrophils for engendering stem-like function in CT26 parental cells by interleukin-1β expression. **a** Immunoblotting of KC (CXCL1) and MIP-1 (CXCL2) in CRC cells. **b** Transmigration assay of neutrophils. IgG, normal IgG (10 μg/ml); CXCL1 nAb, neutralizing antibody against CXCL1 (5 μg/ml); CXCL2 nAb, neutralizing antibody against CXCL2 (5 μg/ml). *P < .05, ***P < .001. **c** RT-qPCR examining IL-1β expression in SDCSC exosome-trained neutrophils. Control CM, complete DMEM medium; CT26 CM, condition medium of parental CT26 cells. The representative results were from two independent assays. *P < .05. **d** A histogram showing spheroid forming capacity of parental CT26 cells. PBS-CM, condition medium from PBS-treated neutrophils; SDCSC-Ex-CM, condition medium from SDCSC exosome-treated neutrophils; IgG, normal IgG (10 μg/ml); IL-1β nAb, Il-1b neutralizing antibody (10 μg/ml). *P < .05, **P < .01. **e** The tumorigenicity of CT26 cells cultured under condition medium of neutrophils. Scale bar = 1 cm. **f** Upper: timeline for showing the sequence of antibody treatment and tumor inoculation. Red arrow, antibody treatment. Lower: a histogram for showing circulating neutrophil concentration at day 0 upon antibody treatment in tumor-free mice. **P < .01. **g** Representative images of tumors. Scale = 1 cm. **h** A histogram illustrating tumor weight of harvested tumors. *P < .05. **i** Expression of *MPO* in CRCSC signaling on (SNAI1^+^/IL8^+^) and off (SNAI1^−^/IL8^−^) CRC patients. ***P < .001. The expression profile of GDC TCGA COAD dataset was used for analysis. **j** A Kaplan-Meier plotter for showing overall survival of CRC patient with 10% top and bottom *MPO* expression. **k** The schematic representation of multistep CRCSC-neutrophil interaction for tumor progression

Though SDCSC exosomes promoted the survival of bone marrow-derived neutrophils, direct SDCSC exosome administration via the tail vein injection did not lead to neutrophil mobilization away from the bone marrow (Additional file 2: Figure S2C), suggesting a localized effect of CRCSC exosomes on neutrophils in the bone marrow. Other CRCSC-secreted components may contribute to systemic neutrophil distribution. Because binding of CXCR2 and CXC chemokines, including CXCL1 (keratinocyte-derived chemokine, KC) and CXCL2 (macrophage inflammatory protein-2, MIP-2), is important for neutrophil recruitment [32, 33], we then examined the expression of CXC chemokines in CRCSCs. Increased expression of CXCL1 and CXCL2 in CT26-SDCSCs was confirmed by western blotting (Fig. 6a). The elevated Transwell migration ability of SDCSC exosome-stimulated neutrophils was decreased upon blocking of CXCR2 ligands by neutralizing CXCL1 and CXCL2 in conditioned medium from CT26-SDCSCs (Fig. 6b and Additional file 5: Figure S4A-B). Additionally, it was found that the expression of *Il1b* was elevated in SDCSC exosome-educated neutrophils when cultured in conditioned medium from CT26 parental cells (Fig. 6c). Neutralization of IL-1β reduced the neutrophil-induced spheroid formation capacity and tumorigenesis of CT26 cells (Fig. 6d, e, respectively).

If neutrophils permit the pro-tumoral host environment, targeting neutrophils may benefit tumor eradication. To examine this notion, we utilized a Ly6G-specific antibody (clone 1A8) to deplete neutrophils and investigated the tumorigenesis of CRCSCs. We found that the circulating neutrophil concentration was reduced 4 days after the initial Ly6G antibody injection in healthy mice (Fig. 6f). Reduced tumor volume of SDCSCs was observed in tumor-bearing mice receiving an Ly6G antibody injection every 4 days (Fig. 6g, h), confirming the critical role of neutrophils for outgrowth of CRCSCs.

### Increased expression of the neutrophil marker *MPO* in CRC patients with a SNAI1+/IL8+ CRCSC profile

We previously demonstrated that Snail activates IL8 expression to maintain the expression of embryonic stem cell genes and self-renewal of CRC patient-derived cancer spheroids [19]. Coexpression of Snail and IL8 is closely related to expression of the CSC marker, CD44 [19]. Here, we found that CRC patients with a CRCSC activation pattern (SNAI1+/IL8+) showed increased *MPO* expression (a neutrophil marker) (Fig. 6i) and high expression of *MPO* predicted poor patient survival (Fig. 6j) in a TCGA dataset. We summarized our findings in Fig. 6k. In CRCSC-dominant primary tumors, CRCSC exosome secretion is increased, and the exosomes are transported to the bone marrow, where they extend neutrophil survival via exosomal tri-phosphate RNAs to activate PRR-NF-κB signaling and IL-1β expression (distal effect, the first tumor-host interaction). Secretion of CRCSC chemokines then helps in the recruitment of exosome-trained neutrophils to primary tumors (proximal effect, the second tumor-host interaction) to accelerate tumorigenesis induced by IL-1β.

## Discussion

The activation of pattern recognition receptors (PRRs) through recognition of pattern-associated molecular patterns (PAMPs) associated with microbial pathogens and damage-associated molecular patterns (DAMPs) associated with cellular components during damage, infection, and stress conditions is critical for initiating the innate immune response [34, 35]. Here, we report that tumor exosomal 5′-triphosphate RNA is a transportable cellular molecular pattern that contributes to expansion of the neutrophil pool for primary tumor infiltration.

Given that let-7-loaded microvesicles promote increased vulnerability to alcoholic neuropathy by activating TLR7 in neurons [36], exosomal miRNAs may contribute to activation of PRRs and neutrophil survival. We showed that miRNA-146a-5p (miR-146a-5p) is a stem miRNA required for symmetric division of human CRCSCs in a previous study [37]. Intriguingly, the CT26-SDCSCs were miR-146a-5p dominant CRCSCs, the miR-146a/Numb circuit was activated (Additional file 6: Figure S5A-B), and knocking down miR-146a-5p abolished the signaling axis (Additional file 6: Figure S5C) as well as spheroid-forming capacity (Additional file 6: Figure S5D). Increased expression of exosomal miR-146a-5p in expanded murine CRCSCs was also observed (Additional file 7: Table S2), and the expression of miR-146a-5p increased in neutrophils upon SDCSC exosome treatment (Additional file 8: Figure S6A). Nevertheless, neutralizing CRCSC exosome-loaded miR-146a-5p by a miR-146a antagomiR had no effects on the survival of host neutrophils (Additional file 8: Figure S6B-C), indicating that exosomal RNA and cellular context are related and necessary to execute biological functions in recipient cells.

The immunosuppressive microenvironment in localized tumors not only enhances tumor progression but also hampers efficient tumor immunotherapy. Myeloid-derived suppressor cells (MDSCs) represent a heterogeneous population of myeloid cells encompassing myeloid progenitors, neutrophils, and monocytes and exhibit immunosuppression in tumor-bearing mice [38]. Tumor-promoting inflammation is emerging as a therapeutic target and one of the hallmarks of cancer [39], and prolonged stimulation with inflammatory cytokines, including IL-1β, has been implicated in the generation and accumulation of MDSCs [40, 41]. In tumor-bearing mice, tumor-associated neutrophils (TANs) share features of granulocytic MDSCs (G-MDSCs) and both are polymorphonuclear Ly6G^+^/ Ly6C^low^ immunosuppressive cells. An increased number of tumor-infiltrating neutrophils is also correlated with malignant phenotypes in patients with solid tumors [42, 43].

To investigate the immunosuppressive potential of CRCSC exosome-stimulated neutrophils, we cultured CD3/CD28 bead-activated splenic CD4T cells in conditioned medium from SDCSC exosome- or PBS-treated neutrophils. It was found that conditioned medium from SDCSC exosome-trained neutrophils suppressed the proliferation of activated T cells (Additional file 9: Figure S7A, right panel) and attenuated the expression of *Il2*, a key cytokine for T cell proliferation [44], and *Ifnr*, a mediator of T cell-dependent anti-tumor responses [45], in activated T cells (Additional file 9: Figure S7B). Moreover, the SDCSC exosome-activated neutrophil signature was positively correlated with the global profile of G-MDSCs and TANs (Additional file 9: Figure S7C). In contrast, direct treatment of CD3/CD28-activated splenocytes with SDCSC exosomes did not influence their proliferation (Additional file 9: Figure S7D), indicating critical roles of CRCSC exosome-educated neutrophils in T cell suppression.

IL-1β is a pleiotropic proinflammatory cytokine associated with diverse diseases, and accumulating evidence supports multifaceted roles of IL-1β in immune modulation and cancer progression. During blood myeloid regeneration, administration of IL-1β promotes myeloid differentiation of HSCs and accelerates myeloid yields following acute injury of the bone marrow [46]. In cancer, an enhanced accumulation of MDSCs, which facilitated tumor progression, was noted in mice with tumors derived from IL-1β-secreting fibrosarcoma cells [47] and 4T1 cells [48]. Stomach-specific expression of IL-1β in transgenic mice further lead to gastric dysplasia and MDSC accumulation in the stomach [41]. Inhibition of IL-1β activity prevents tumor invasiveness [49] and metastatic colonization [50]. Enhanced expression of TNF-α in CRCSC exosome-stimulated neutrophils (Additional file 4: Figure S3C) and induction of TNF-α by IL-1β [51] may contribute to activation-induced cell death in T cells [52]. Here, we expand the present understanding of IL-1β and demonstrate a dual role of neutrophil-secreted IL-1β in extension of neutrophil survival and promotion of spheroid forming capacity of CRC cells.

The number of neutrophils homeostasis is maintained via two independent manners. First, the HSCs/myeloid progenitors are critical sources for maintaining number of differentiated myeloid cells including neutrophils through differentiation. For example, high levels of IL-1β produced by monocytes and endothelial cells in bone marrow microenvironment instructs HSCs toward biased myeloid differentiation during bone marrow injury through the activation of NF-κB pathway and a PU.1-dependent myeloid gene remodeling [46]. Second, the environmental stress and external signal are able to regulate the number of differentiated myeloid cells aside from HSCs. For example, under hypoxic microenvironments, HIF-1α extends the survival of hypoxic neutrophil by activating macrophage inflammatory protein-1β (MIP-1β) [53]. Furthermore, glucocorticoids-induced upregulation of LTB4 receptor BLT1 enhances the anti-apoptotic effect of LTB4 in neutrophils [54]; IL-1β has been known to prolong the lifespan of neutrophil [55, 56]. In this study, we demonstrated CRCSCs exosome-mediated IL-1β expression in neutrophils prolonged neutrophil survival (Fig. 5b, c), which establishes the host-tumor crosstalk for facilitating tumorigenesis through extending the survival pro-tumoral neutrophils.

Because most neutrophils reside in the bone marrow and less than 2% of neutrophils are found in peripheral blood [28], chemotaxis of neutrophils to infected lesions or localized tumors is fundamental for their function. In this study, direct administration of SDCSC exosomes elevated the neutrophil population in the bone marrow (Fig. 4f) but not their peripheral accumulation in the spleen (Additional file 2: Figure S2C), suggesting that CRCSC chemokines may contribute to neutrophil recruitment. In humans, IL8 serves as a central chemokine for recruitment of neutrophils and G-MDSCs [57, 58]. We previously identified that the epithelial-to-mesenchymal transition (EMT) regulator Snail was a predominant EMT transcription factor in CRCSCs that activated IL8 expression directly to maintain stem-like features [19]. A recent study also showed that Snail activates CXCR2 ligand expression to recruit MDSCs for ovarian cancer progression [59]. Tumor infiltrating neutrophils further increase tumor hypoxia and stabilize Snail expression [60]. Here, the expression of MPO (a neutrophil marker) was found to be elevated in CRC patients with a SNAI1^+^/IL8^+^ CRCSC activation pattern (Fig. 6i), suggesting the involvement of neutrophils in mediating EMT and CRCSC niches.

## Conclusions

Altogether, our research describes the heterogeneity of tumor exosomes and elucidates a unique behavior of tumor exosomal RNAs in the establishment of a pro-tumoral microenvironment through expansion of host myeloid cells prior to their blood emergence and tumor recruitment. Strategies to monitor tumor-host interactions by examining circulating tumor exosomes will provide crucial information necessary for adjusting immuno-oncology therapies in cancer patients.

## Additional files

Additional file 1: **Figure S1.** Characterization of SCCSC-exosome-stimulated neutrophils. (A) The flow cytometry results illustrating uptake of pHrodo Re-labeled E-coli of neutrophils. (B) A flow cytometry result for showing ROS activity of neutrophils upon treatment. PBS control, PBS treatment; Exosome control, treatment of 20 μg/ml of SDCSC- exosomes; DCFDA, the DCFDA stained cells, Treatment of TBHP was utilized as a positive control. (C) Flow cytometry results for showing expression of CXCR2. (PDF 515 kb)

Additional file 2: **Figure S2.** Effects of SDCSC-exosomes on hematopoietic stem cells, progenitors and neutrophils. (A) The flow cytometry results for showing gating strategies for mouse hematopoietic stem cells and progenitors. LSK cells, lineage(-)ScaI(+)Kit(+) hematopoietic stem cells; LK, lineage(-)Kit(+) progenitors; GMPs, granulocyte-macrophage progenitors; CMPs, common myeloid progenitors; MEPs, megakaryocyte-erythroid progenitors, Lin, lineage marker. (B) Percentage of LSK, GMPs, CMPs and MEPs in bone marrows of mice injected with PBS (*N*=4) or CT26-SDCSC-exosomes (*N*=5). A total of 45 μg exosomes were injected. Data represent the mean ±S.D. (C) Percentage of neutrophils in spleens of mice injected with PBS (*N*=4) and CT26-SDCSC-exosomes (*N*=5). A total of 45 μg of SDCSC-exosomes were injected through tail vein. Data represent the mean ± SEM. (PDF 295 kb)

Additional file 3: **Table S1.** The gene expression profile of SDCSC-exosome-treated neutrophils by RNA-seq. (XLS 392 kb)

Additional file 4: **Figure S3.** Gene ontology analysis of SDCSC-exosome-treated neutrophils. (A) A histogram showing the enriched disease and biological categories from 3056 genes activated in SDCSC-exosome-trained neutrophils based on Ingenuity Pathway Analysis (IPA). (B) A histogram for illustrating enriched canonical signaling pathways of SDCSC-stimulated neutrophils with IPA. (C) The connectivity network established from genes in Top 2 categories of (B). (PDF 4166 kb)

Additional file 5: **Figure S4.** The trans-well migration potential of SDCSC-exosome-treated neutrophils. (A) A flow chart illustrating the experimental design for quantifying migrated cells by flow cytometry. (B) Representative results for counting migrated cells by flow cytometry. IgG, normal IgG (10 μg/ml); CXCL1 nAb, neutralizing antibody against CXCL1 (5 μg/ml); CXCL2 nAb, neutralizing antibody against CXCL2 (5 μg/ml). (PDF 2541 kb)

Additional file 6: **Figure S5.** Examination of miR-146a-5p/Numb axis in expanded CT26-SDCSCs. (A) RT-qPCR of cellular miR-146a-5p expression. Data represent the mean ± S.D. **P<.01. (B) Immunoblots for showing expression of Numb and β-catenin. (C) RT-qPCR of cellular miR-146a-5p and Numb expression upon silencing miR-146a-5p. Zc, a shRNA control; Z146a, a shRNA targeting miR-146a-5p. (D) Spheroid-forming capacity upon silencing miR-146a-5p in CT26-SDCSCs. Data represent the mean ± S.D. ***P<.001. (PDF 242 kb)

Additional file 7: **Table S2.** The differentially expressed small non-coding RNAs in SDCSC-exosomes. (XLS 178 kb)

Additional file 8: **Figure S6.** Effects of SDCSC-exosomal miR-146a-5p on survival of neutrophils. (A) RT-qPCR examining expression of cellular miR-146a-5p upon SDCSC-exosome administration. Data represent the mean ± S.D. **P<.01. (B) A histogram showing relative viability of neutrophil receiving antogomiRs. Control antagomiR, 200 nM of cel-miR-67-3p antagomiR; miR-146a antagomiR, 200 nM of miR-146a-5p antagomiR. Cells were transfected with indicated antagomiRs for 4 hours followed by SDCSC-exosome treatment for 3 days. Data represent the mean ± S.D. (C) RT-qPCR validation of miR-146a-5p expression in cells from (B). Data represent the mean ± S.D. **P<.01. (PDF 405 kb)

Additional file 9: Figure S7. Acquisition of an immunosuppressive phenotype in bone marrow-derived neutrophils upon administration of SDCSC-exosomes. (A) T-cell suppression assay. Left: histogram showing proliferation of CD4-T cells treated with PBS or CD3/CD28 beads. Data represent the mean ± S.D. *P<.05. Right: histogram showing the proliferation of CD4-T cells treated with CD3/CD28 beads under indicated conditions. PBS, PBS-treated; Beads, CD3/CD28 bead; CM beads, CD4-T cells were treated with condition medium from PBS-treated neutrophil in the presence of CD3/CD28 beads; SDCSC Ex-CM beads, T-cells were treated with condition medium from SDCSC-exosome-treated neutrophils in the presence of CD3/CD28 beads. Data represent the mean ± S.D. *P<.05. (B) RT-qPCR under the indicated conditions. CM, CD4-T cells treated with condition medium from PBS-treated neutrophils; CM beads, CD4-T cells treated with condition medium from PBS-treated neutrophils with CD3/CD28 beads; SDCSC-Ex-CM beads, CD4-T cells treated with condition medium from CT26-SDCSC-exosome-treated neutrophil and with CD3/CD28 beads. Data represent the mean ± S.D. *P<.05. (C) GSEA results showing the association between the top 500 SDCSC-activated neutrophil signature and G-MDSC (left panel) or TAN (right panel) profiles. The expression profiles of TAN, G-MDSC and BM-Neu were from GSE43254. ES, enrichment score; NES, nominal enrichment score; FDR, false discovery rate; TAN, tumor-infiltrated neutrophil; G-MDSCs, granulocytic myeloid-derived suppressor cells; BM-Neu, bone marrow-derived neutrophils. (D) The relative viability of CD3/CD28-activated splenocytes. Splenocytes were treated with 20 μg/ml of CT26-SDCSC-exosomes or PBS in the presence of CD3/CD28 beads for 72 h. Data represent the mean ± S.D. (PDF 490 kb)

## Abbreviations

CRC: Colorectal carcinoma
CRCSCs: Colorectal cancer stem cells
CSCs: Cancer stem cells
EMT: Epithelial-to-mesenchymal transition
FBS: Fetal bovine serum
GMPs: Granulocyte-macrophage progenitors
ILVs: Intraluminal vesicles
ISCs: Intestinal stem cells
LINE: Long interspersed nuclear elements
LSK-HSCs: Lineage marker (−)/ScaI (+)/Kit (+) hematopoietic stem cells
LTR: Long terminal repeat
MDSC: Myeloid-derived suppressor cells
MEPs: Megakaryocyte-erythroid progenitors
miRNA: microRNA
PRRs: Pattern recognition receptors
scRNA: Small cytoplasmic RNA
SDCSCs: Sphere-derived cancer stem cells
SINE: Short interspersed nuclear elements
snoRNA: Small nucleolar RNA
snRNA: Small nuclear RNA
srpRNA: Signal recognition particle RNA
TEM: Transmission electron microscopy
TME: Tumor microenvironment
tRNA: Transfer RNA
