# Supplementary figures and images for: Tumor stem-like cell-derived exosomal RNAs prime neutrophils for facilitating tumorigenesis of colon cancer
Source: J Hematol Oncol. 2019 Jan 25;12:10. doi: 10.1186/s13045-019-0699-4 (PMC6347849; doi:10.1186/s13045-019-0699-4)

A

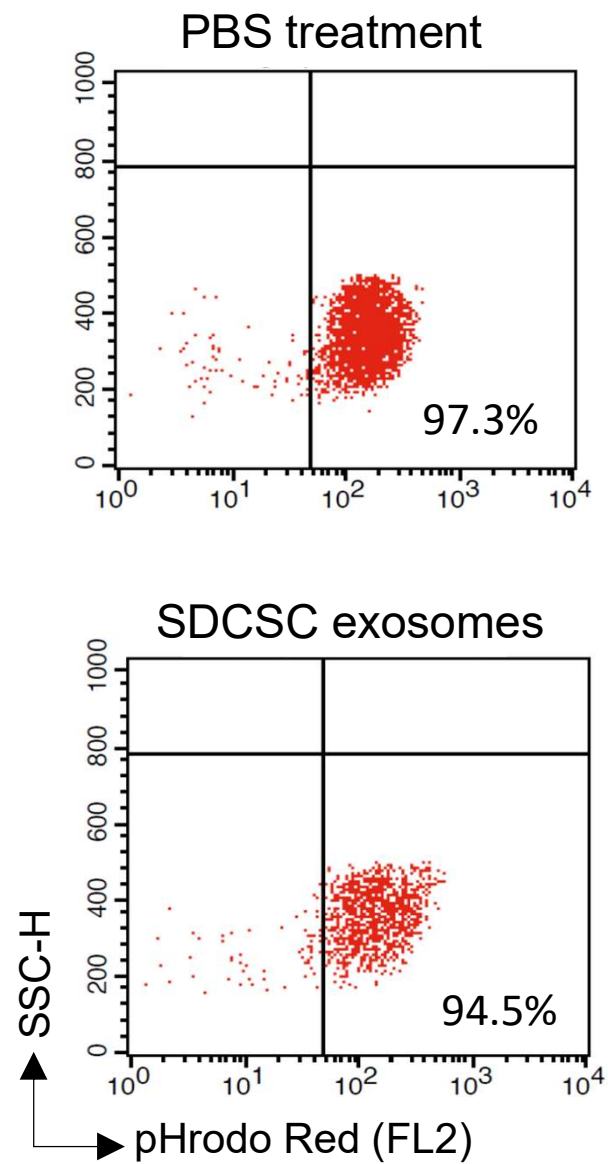

B

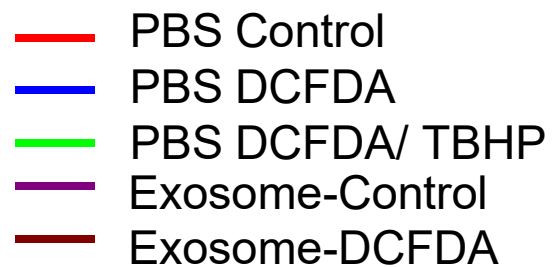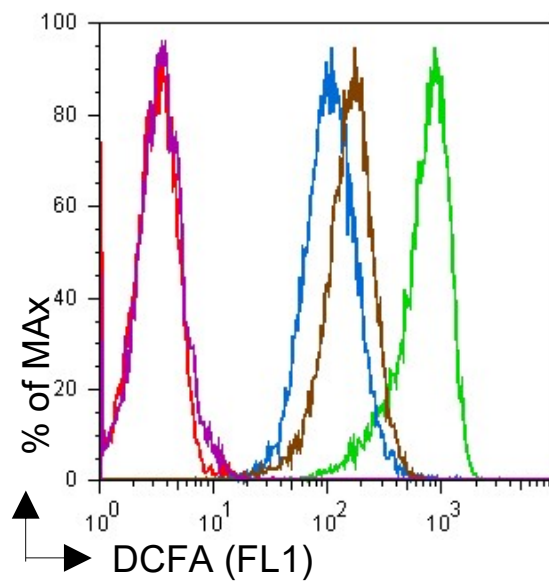

C

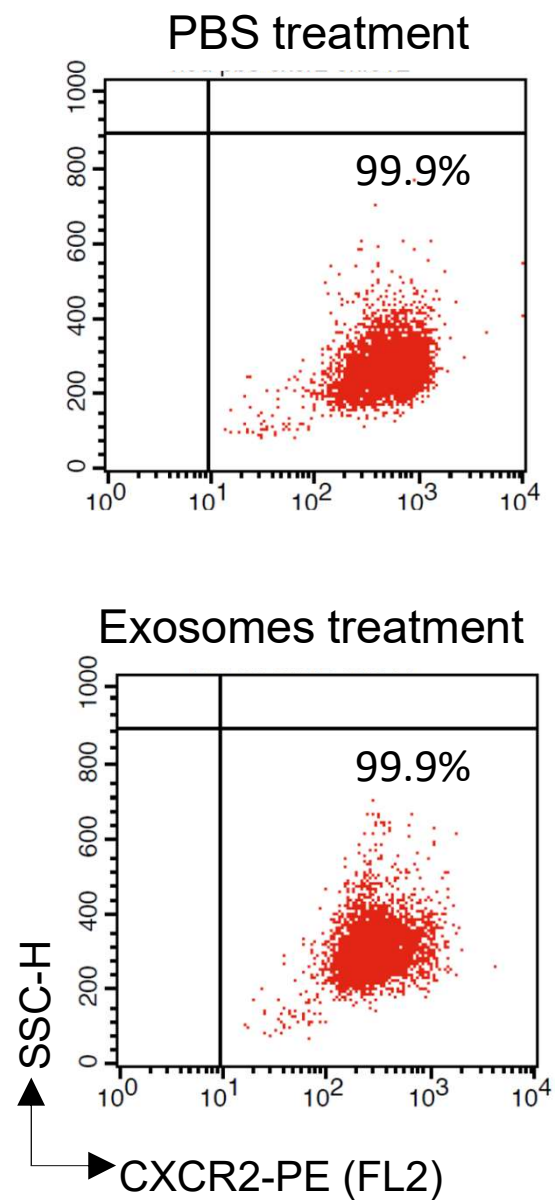

Supplement: Supplementary file 1 — Figure S1. Characterization of SCCSC-exosome-stimulated neutrophils. (A) The flow cytometry results illustrating uptake of pHrodo Re-labeled E-coli of neutrophils. (B) A flow cytometry result for showing ROS activity of neutrophils upon treatment. PBS control, PBS treatment; Exosome control, treatment of 20 μg/ml of SDCSC- exosomes; DCFDA, the DCFDA stained cells, Treatment of TBHP was utilized as a positive control. (C) Flow cytometry results for showing expression of CXCR2. (PDF 515 kb) [file 13045_2019_699_MOESM1_ESM.pdf]

A

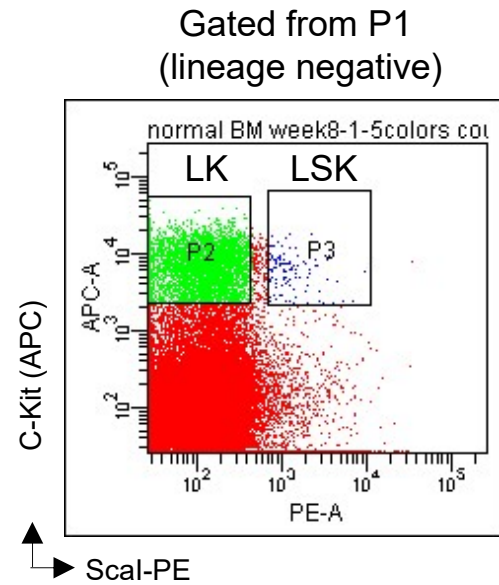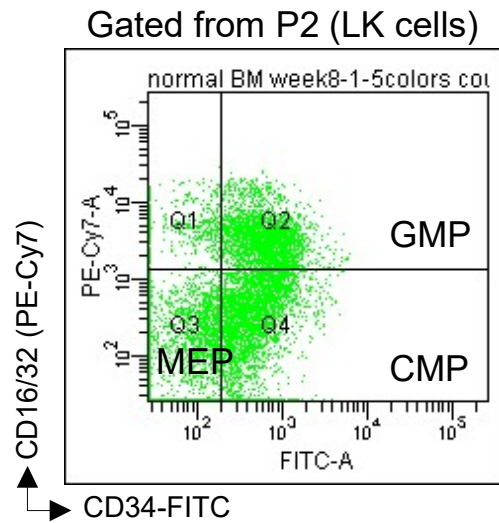

B

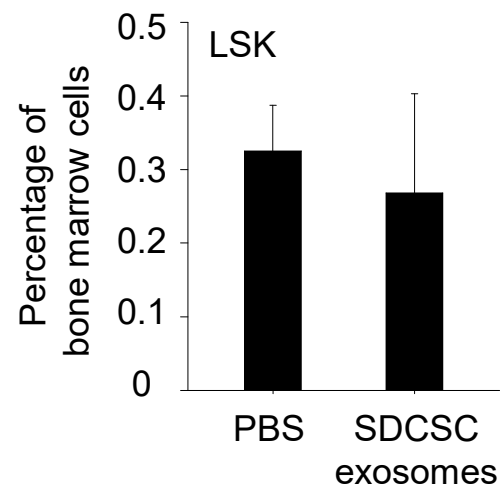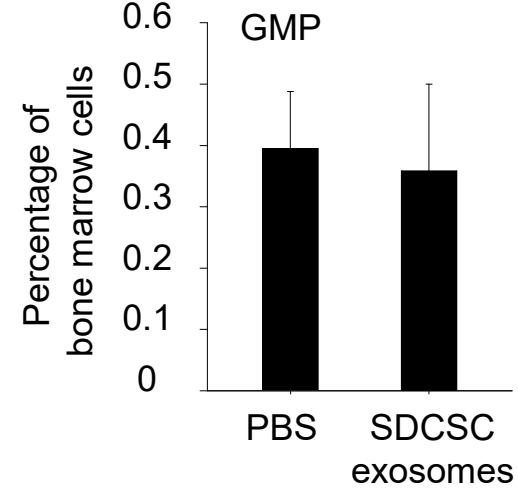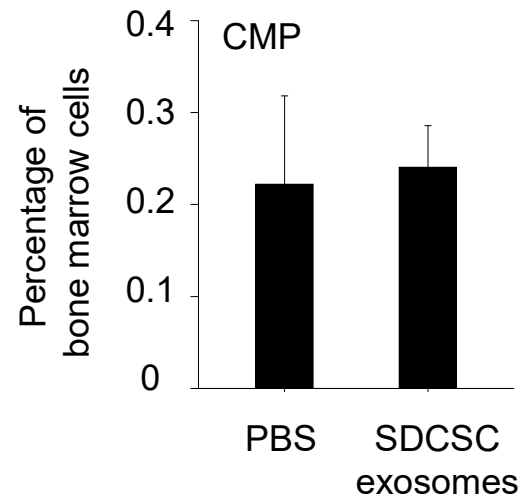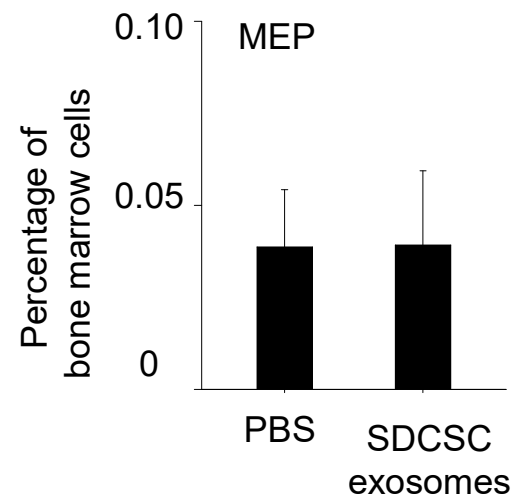

C

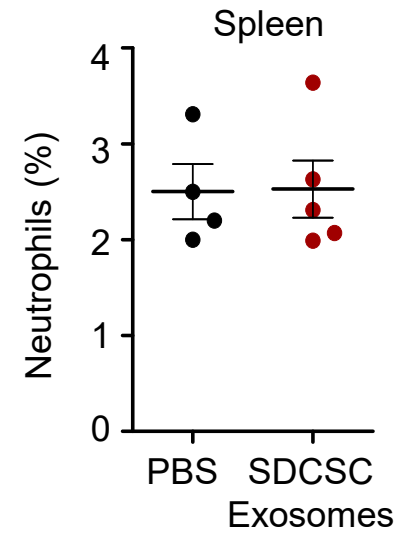

Supplement: Supplementary file 2 — Figure S2. Effects of SDCSC-exosomes on hematopoietic stem cells, progenitors and neutrophils. (A) The flow cytometry results for showing gating strategies for mouse hematopoietic stem cells and progenitors. LSK cells, lineage(-)ScaI(+)Kit(+) hematopoietic stem cells; LK, lineage(-)Kit(+) progenitors; GMPs, granulocyte-macrophage progenitors; CMPs, common myeloid progenitors; MEPs, megakaryocyte-erythroid progenitors, Lin, lineage marker. (B) Percentage of LSK, GMPs, CMPs and MEPs in bone marrows of mice injected with PBS (N=4) or CT26-SDCSC-exosomes (N=5). A total of 45 μg exosomes were injected. Data represent the mean ±S.D. (C) Percentage of neutrophils in spleens of mice injected with PBS (N=4) and CT26-SDCSC-exosomes (N=5). A total of 45 μg of SDCSC-exosomes were injected through tail vein. Data represent the mean ± SEM. (PDF 295 kb) [file 13045_2019_699_MOESM2_ESM.pdf]

**A**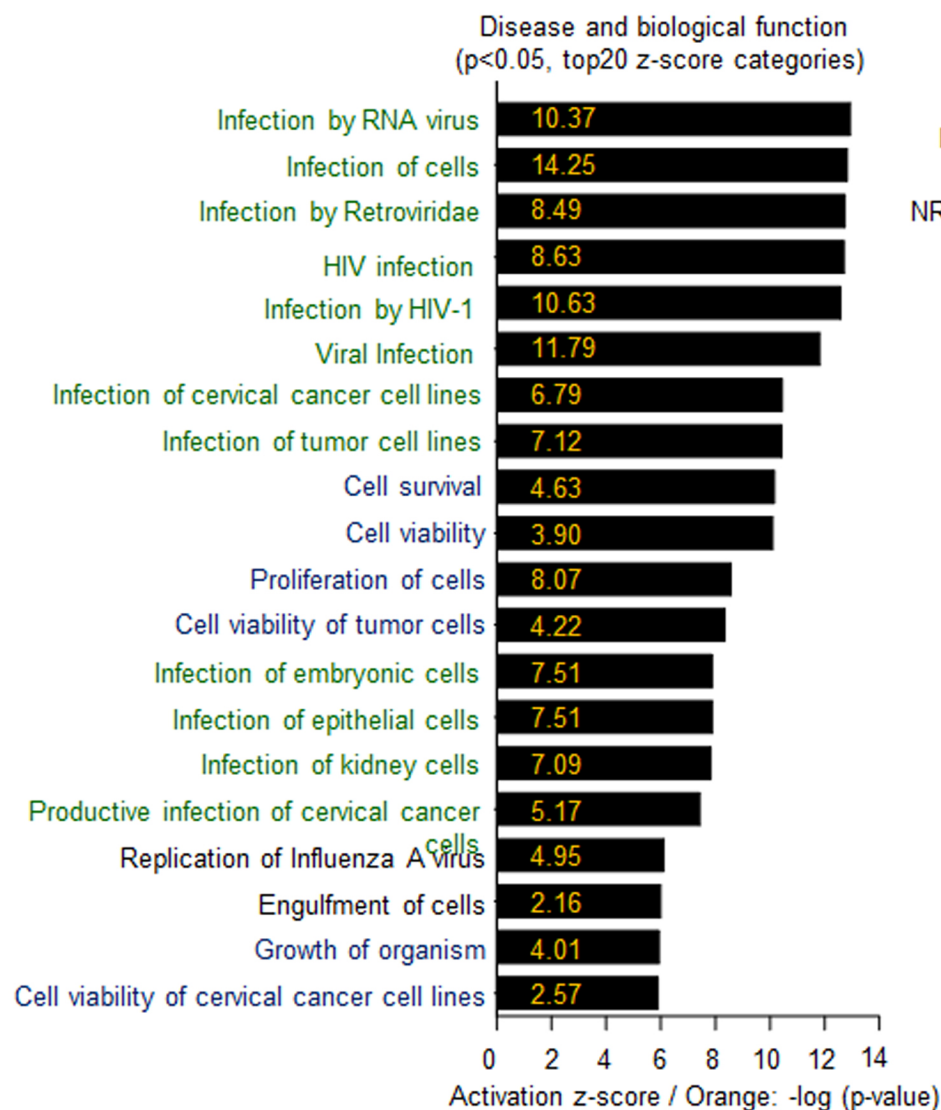**B**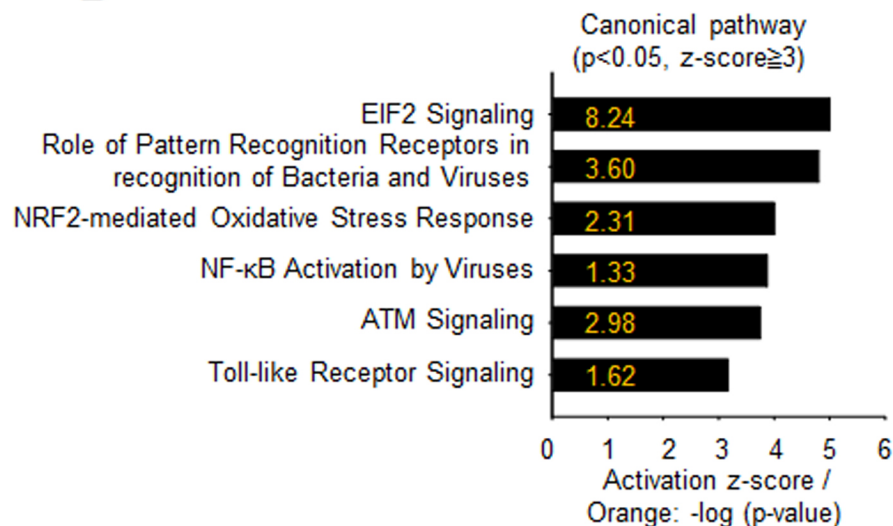**C**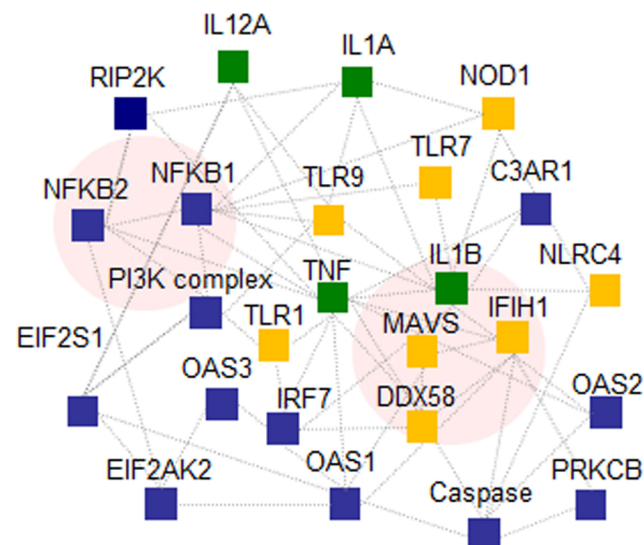

Supplement: Supplementary file 4 — Figure S3. Gene ontology analysis of SDCSC-exosome-treated neutrophils. (A) A histogram showing the enriched disease and biological categories from 3056 genes activated in SDCSC-exosome-trained neutrophils based on Ingenuity Pathway Analysis (IPA). (B) A histogram for illustrating enriched canonical signaling pathways of SDCSC-stimulated neutrophils with IPA. (C) The connectivity network established from genes in Top 2 categories of (B). (PDF 4166 kb) [file 13045_2019_699_MOESM4_ESM.pdf]

**A**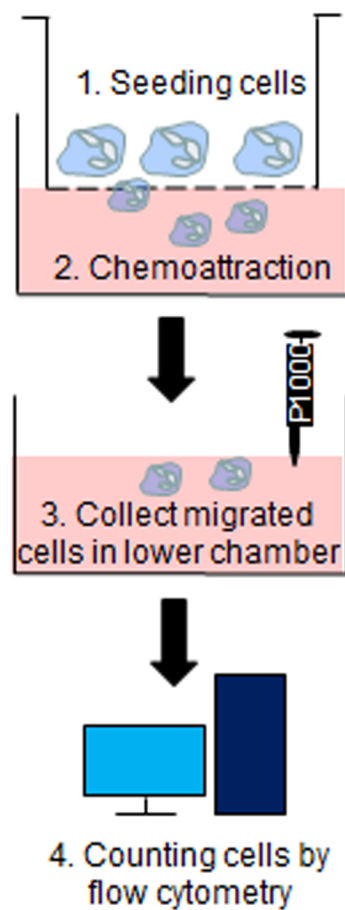**B**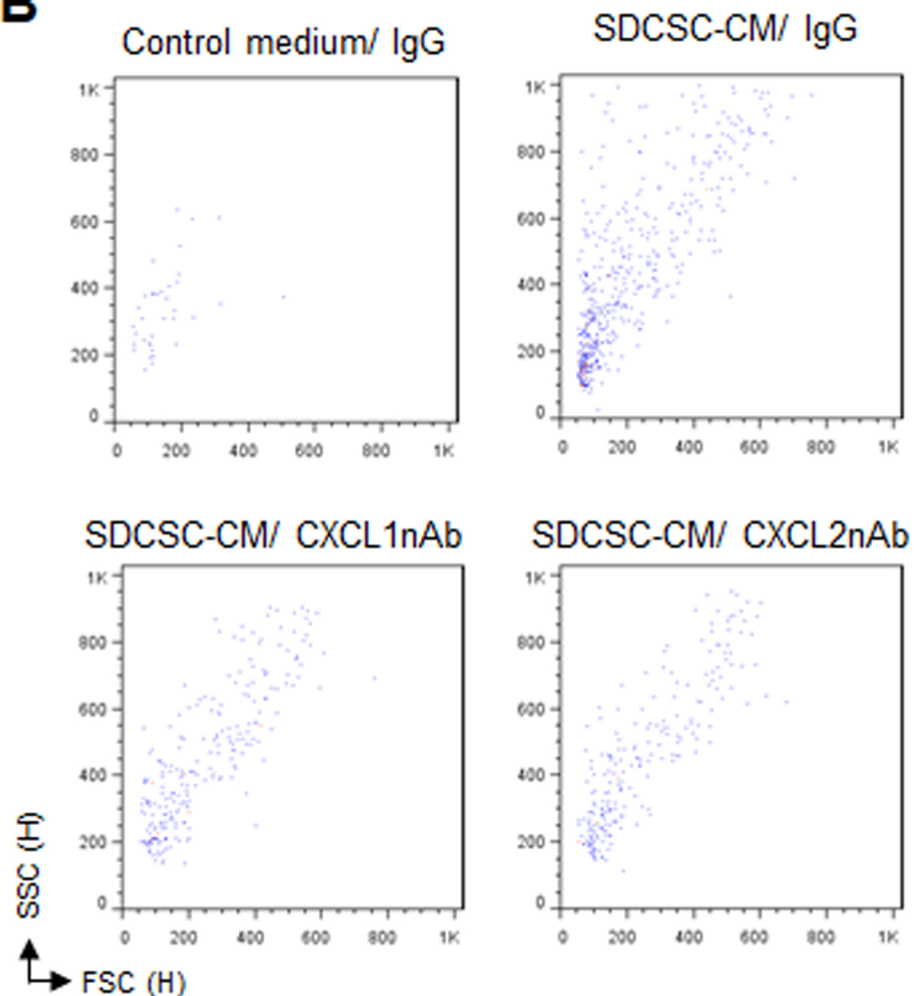

Supplement: Supplementary file 5 — Figure S4. The trans-well migration potential of SDCSC-exosome-treated neutrophils. (A) A flow chart illustrating the experimental design for quantifying migrated cells by flow cytometry. (B) Representative results for counting migrated cells by flow cytometry. IgG, normal IgG (10 μg/ml); CXCL1 nAb, neutralizing antibody against CXCL1 (5 μg/ml); CXCL2 nAb, neutralizing antibody against CXCL2 (5 μg/ml). (PDF 2541 kb) [file 13045_2019_699_MOESM5_ESM.pdf]

**A**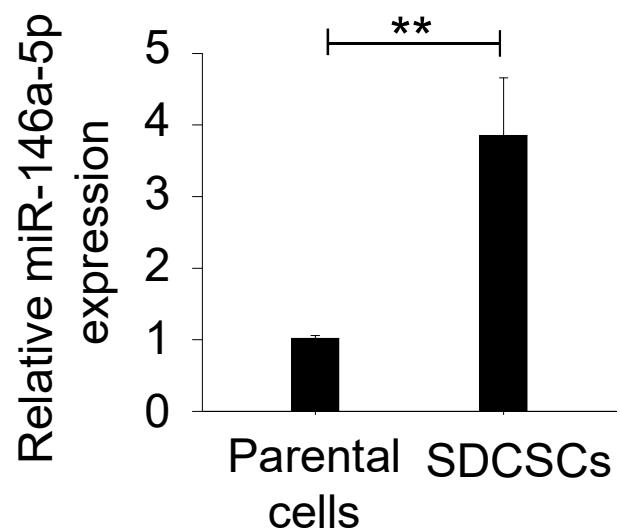**B**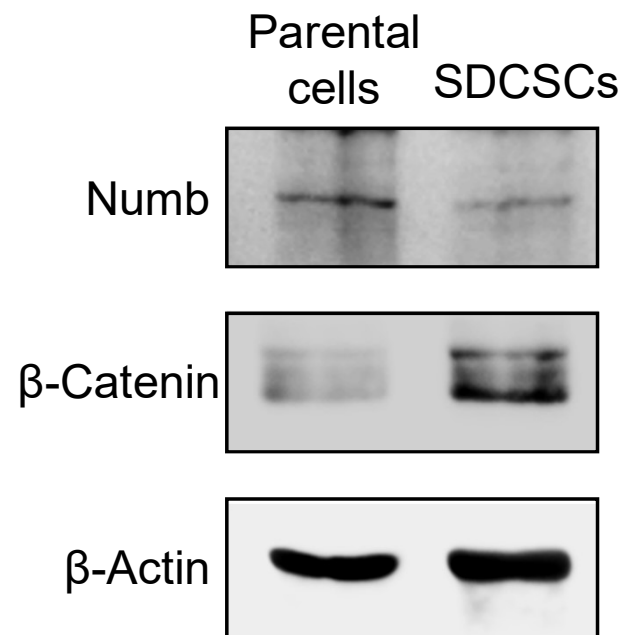**C**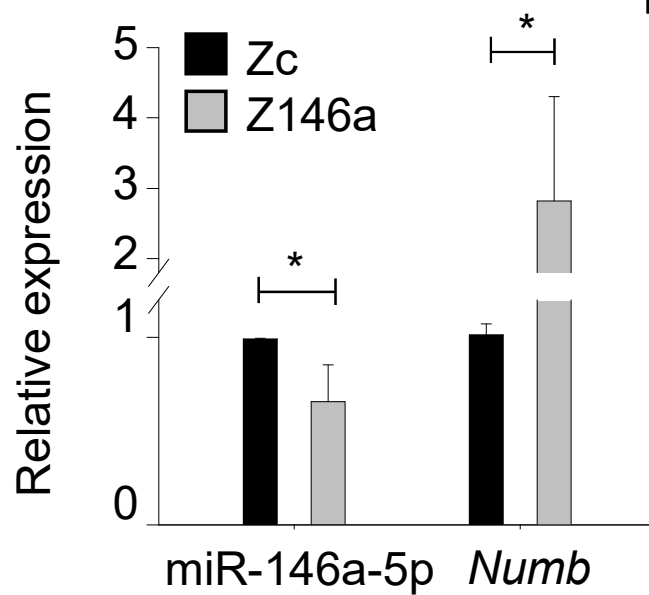**D**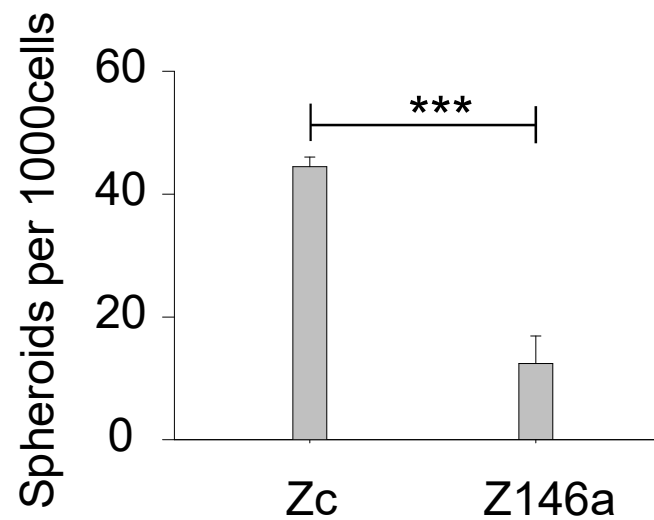

Supplement: Supplementary file 6 — Figure S5. Examination of miR-146a-5p/Numb axis in expanded CT26-SDCSCs. (A) RT-qPCR of cellular miR-146a-5p expression. Data represent the mean ± S.D. **P<.01. (B) Immunoblots for showing expression of Numb and β-catenin. (C) RT-qPCR of cellular miR-146a-5p and Numb expression upon silencing miR-146a-5p. Zc, a shRNA control; Z146a, a shRNA targeting miR-146a-5p. (D) Spheroid-forming capacity upon silencing miR-146a-5p in CT26-SDCSCs. Data represent the mean ± S.D. ***P<.001. (PDF 242 kb) [file 13045_2019_699_MOESM6_ESM.pdf]

A

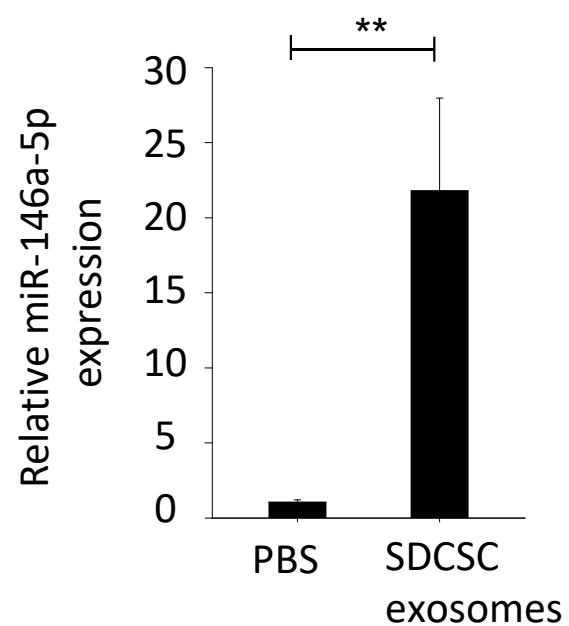

B

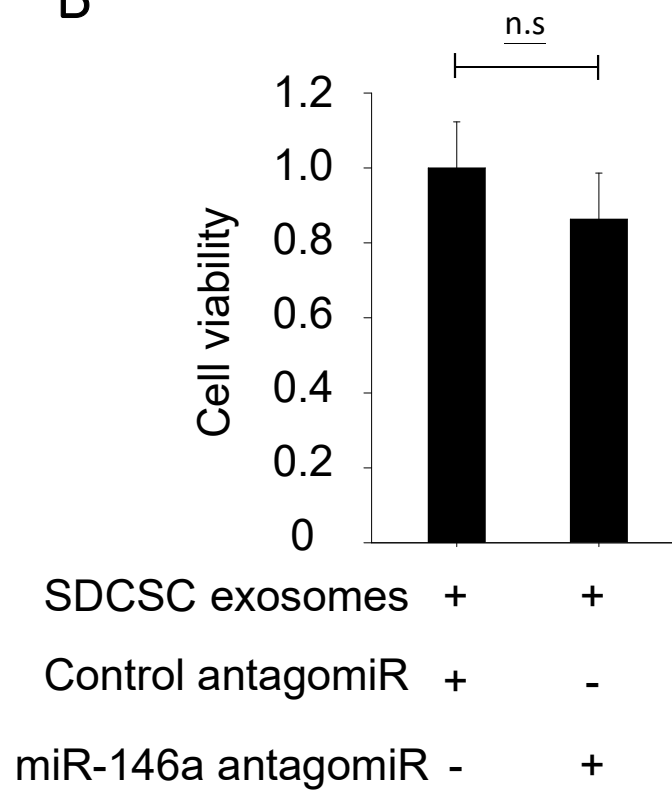

C

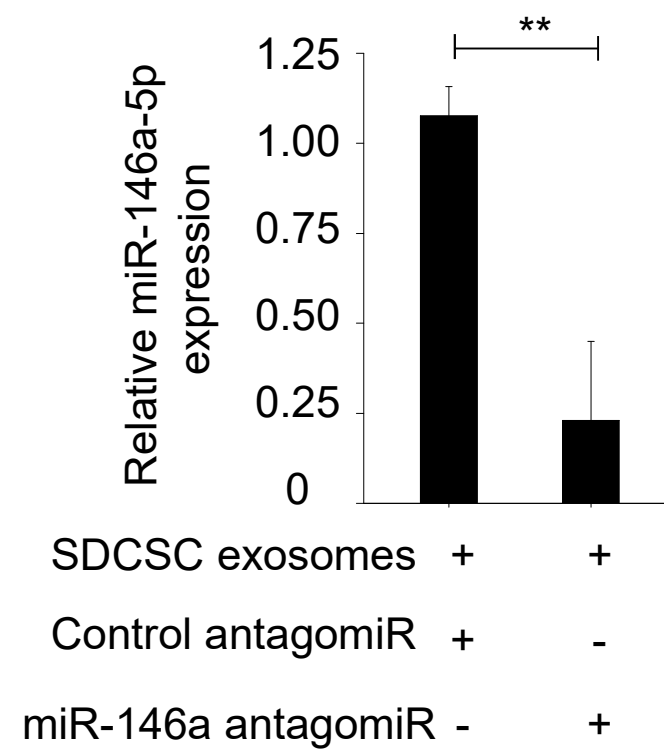

Supplement: Supplementary file 8 — Figure S6. Effects of SDCSC-exosomal miR-146a-5p on survival of neutrophils. (A) RT-qPCR examining expression of cellular miR-146a-5p upon SDCSC-exosome administration. Data represent the mean ± S.D. **P<.01. (B) A histogram showing relative viability of neutrophil receiving antogomiRs. Control antagomiR, 200 nM of cel-miR-67-3p antagomiR; miR-146a antagomiR, 200 nM of miR-146a-5p antagomiR. Cells were transfected with indicated antagomiRs for 4 hours followed by SDCSC-exosome treatment for 3 days. Data represent the mean ± S.D. (C) RT-qPCR validation of miR-146a-5p expression in cells from (B). Data represent the mean ± S.D. **P<.01. (PDF 405 kb) [file 13045_2019_699_MOESM8_ESM.pdf]

A

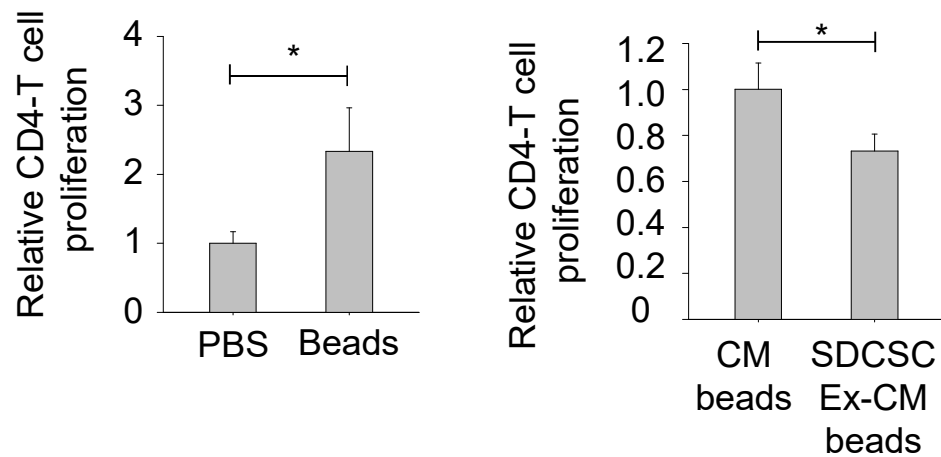

B

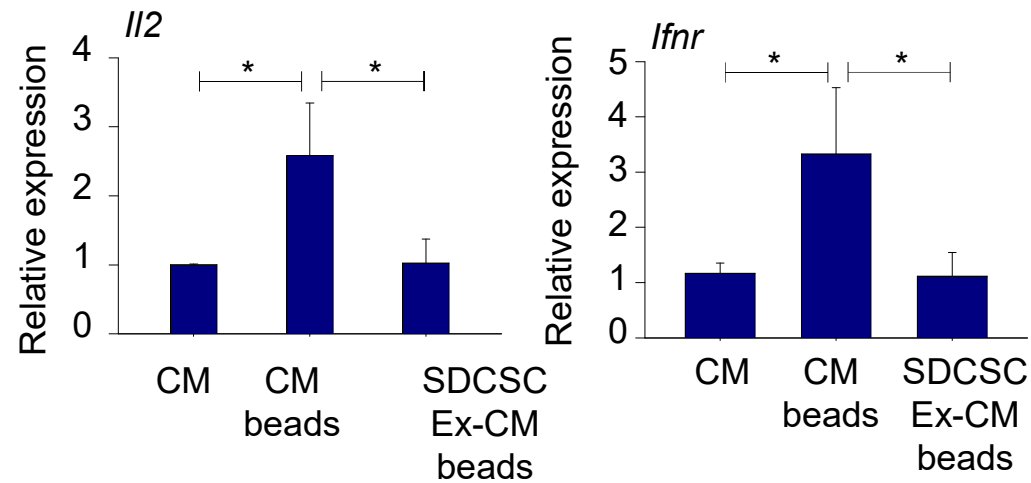

C

Top 500 Neutrophil Signature

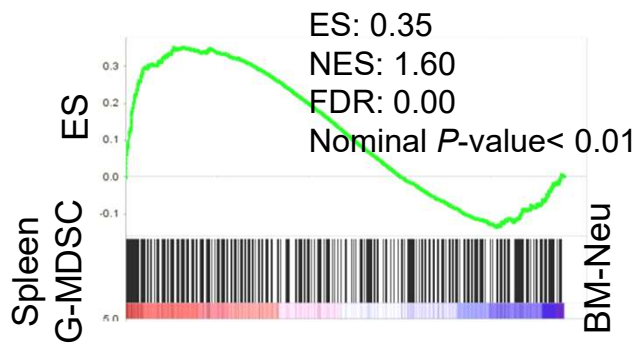

Top500 Neutrophil signature

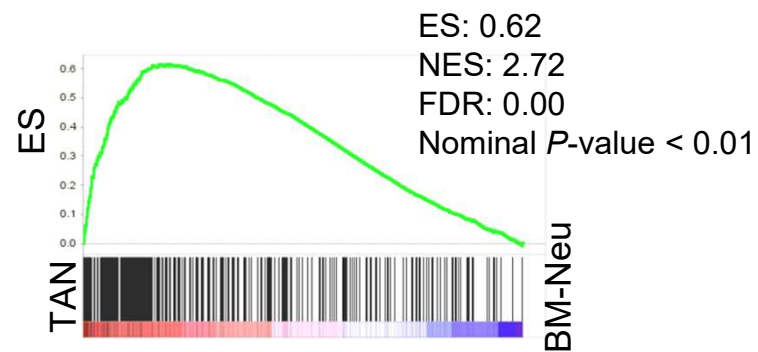

D

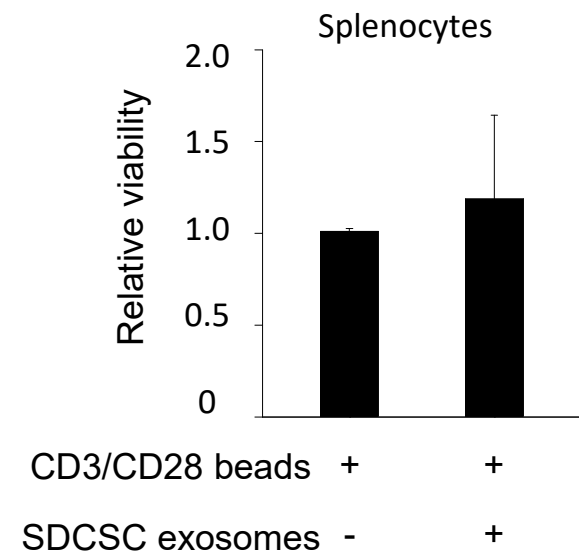

Supplement: Supplementary file 9 — Figure S7. Acquisition of an immunosuppressive phenotype in bone marrow-derived neutrophils upon administration of SDCSC-exosomes. (A) T-cell suppression assay. Left: histogram showing proliferation of CD4-T cells treated with PBS or CD3/CD28 beads. Data represent the mean ± S.D. *P<.05. Right: histogram showing the proliferation of CD4-T cells treated with CD3/CD28 beads under indicated conditions. PBS, PBS-treated; Beads, CD3/CD28 bead; CM beads, CD4-T cells were treated with condition medium from PBS-treated neutrophil in the presence of CD3/CD28 beads; SDCSC Ex-CM beads, T-cells were treated with condition medium from SDCSC-exosome-treated neutrophils in the presence of CD3/CD28 beads. Data represent the mean ± S.D. *P<.05. (B) RT-qPCR under the indicated conditions. CM, CD4-T cells treated with condition medium from PBS-treated neutrophils; CM beads, CD4-T cells treated with condition medium from PBS-treated neutrophils with CD3/CD28 beads; SDCSC-Ex-CM beads, CD4-T cells treated with condition medium from CT26-SDCSC-exosome-treated neutrophil and with CD3/CD28 beads. Data represent the mean ± S.D. *P<.05. (C) GSEA results showing the association between the top 500 SDCSC-activated neutrophil signature and G-MDSC (left panel) or TAN (right panel) profiles. The expression profiles of TAN, G-MDSC and BM-Neu were from GSE43254. ES, enrichment score; NES, nominal enrichment score; FDR, false discovery rate; TAN, tumor-infiltrated neutrophil; G-MDSCs, granulocytic myeloid-derived suppressor cells; BM-Neu, bone marrow-derived neutrophils. (D) The relative viability of CD3/CD28-activated splenocytes. Splenocytes were treated with 20 μg/ml of CT26-SDCSC-exosomes or PBS in the presence of CD3/CD28 beads for 72 h. Data represent the mean ± S.D. (PDF 490 kb) [file 13045_2019_699_MOESM9_ESM.pdf]
